# *Conocybe* Section *Pilosellae* in China: Reconciliation of Taxonomy and Phylogeny Reveals Seven New Species and a New Record

**DOI:** 10.3390/jof9090924

**Published:** 2023-09-13

**Authors:** Han-Bing Song, Tolgor Bau

**Affiliations:** Key Laboratory of Edible Fungal Resources and Utilization (North), Ministry of Agriculture and Rural Affairs, Jilin Agricultural University, Changchun 130118, China; s413637227@163.com

**Keywords:** Bolbitiaceae, *Conocybe* sect. *Pilosellae*, morphology, phylogeny

## Abstract

*Conocybe* belongs to the Bolbitiaceae. The morphological classification and molecular phylogenetics of *Conocybe* section *Pilosellae* are not in agreement. In this study, based on the specimens from China, we investigated the sect. *Pilosellae* and identified 17 species, including 7 new species: *Conocybe pilosa*, with a densely hairy pileus and stipe; *C. reniformis*, with reniform spores; *C. ceracea*, with waxy dehydration of the lamellae; *C. muscicola*, growing on moss; *C. sinobispora*, with two-spored basidia; *C. hydrophila*, with a hygrophanous pileus; *C. rufostipes*, growing on dung with a brown stipe; and *C. pseudocrispa*, one new record for China. A key was compiled for the sect. *Pilosellae* in China. Here, the sect. *Pilosellae*, and new species and records from China are morphologically described and illustrated. Maximum likelihood and Bayesian analyses were performed using a combined nuc rDNA internal transcribed spacer region (ITS) and nuc 28S rDNA (nrLSU), and translation elongation factor 1-alpha (*tef1-α*) dataset to reconstruct the relationships of this section. We found that the sect. *Pilosellae* was the basal clade of *Conocybe*, and its evolutionary features may shed light on the characteristics of *Conocybe*. By integrating morphological classification and phylogenetic analysis, we explored the possible phylogenetic relationships among the species of the sect. *Pilosellae* in China.

## 1. Introduction

The name of *Conocybe* Fay (Basidiomycota, Agaricomycetes, Agaricales, and Bolbitiaceae) originates from the Greek words “konos” and “cybe”, meaning “conical head” and refers to its conical pileus [1]. *Conocybe* is the largest genus in the Bolbitiaceae and is characterized by a conical pileus, brown-rusty lamellae, and slender stipe often covered with powdery or hairy, lecythiform cheilocystidia. *Conocybe* species are widely distributed and often grow in fertile soils and herbivorous dung. They are a group of saprophytic fungi that may contain toxic substances, such as psilocybin, phallotoxins, and amatoxins, which cause neuropsychological problems, gastroenteritis, and liver damage [2]. However, these toxic substances also have potential pharmacological activities and have shown significant therapeutic effects in neurological and psychological disorders, such as treatment-resistant depression and post-traumatic stress disorder [3,4,5].

In 1821, Fries first noticed the *Conocybe* group and established the tribe *Galera*. Since then, the taxonomic status and classification of *Conocybe* have been revised and clarified multiple times. Currently, *Conocybe* is divided into more than 10 sections based on morphology, including the sect. *Conocybe*, sect. *Mixtae*, sect. *Candidae*, and sect. *Pilosellae* [1,6,7,8,9,10,11]. Among them, the sect. *Pilosellae* was first proposed by Kühner (1935) [12], who placed *Pilosellae* in the subgenus *Euconocybe* of the genus *Conocybe*, based on the hairiness and sparsity at the base of the stipe of *Conocybe*. Singer (1949) promoted *Pilosellae* to the sect. *Pilosellae* and placed it within the subgenus *Euconocybe* [13]. Additionally, Singer (1962) formally established the sect. *Pilosellae* based on an incomplete white stipe [14], numerous hairs, and non-lecythiform caulocystidia, and designated *C. pilosella* as the type species. Thereafter, Moser (1978) [8], Watling (1982) [15], Singer (1986) [9], Bon (1992) [16], and Arnolds (2005) [17] revised the classification of *Conocybe* by gradually expanding its definition and adding multiple species to the sect. *Pilosellae*; however, they did not change the taxonomic rank of the sect. *Pilosellae*. Hausknecht (2005) conducted important research on all the European species of the sect. *Pilosellae* and studied related type materials of European and non-European taxa [18]. Hausknecht further revised this section and outlined its characteristics [11]. Limited phylogenetic studies have been conducted on the sect. *Pilosellae*; however, recent studies have clarified its taxonomic status. It has been found that *Conocybe* forms a monophyletic group and the sect. *Pilosellae* belongs to the basal clade of *Conocybe* [19]. These results have been confirmed by Liu (2018) and Ullah et al. (2023) [20,21].

Currently, there are 33 documented species of *Conocybe* in China, of which 13 belong to the sect. *Pilosellae* [20,22,23,24,25,26,27,28,29,30,31,32,33,34]. Most species of the sect. *Pilosellae* are found in the northeastern region of China; however, it is not clear whether they also exist in tropical and subtropical areas. This study was based on specimens collected from the Tianjin, Inner Mongolia, Jilin, Hubei, Hunan, and Guangxi provinces in China as well as Fungarium specimens (Mycology Herbarium of the Jilin Agriculture University (HMJAU)). This study aimed to conduct systematic macroscopic, microscopic, and molecular research on the species of the sect. *Pilosellae* in China.

## 2. Material and Methods

### 2.1. Samplings and Morphological Analyses

**Figure 1:**
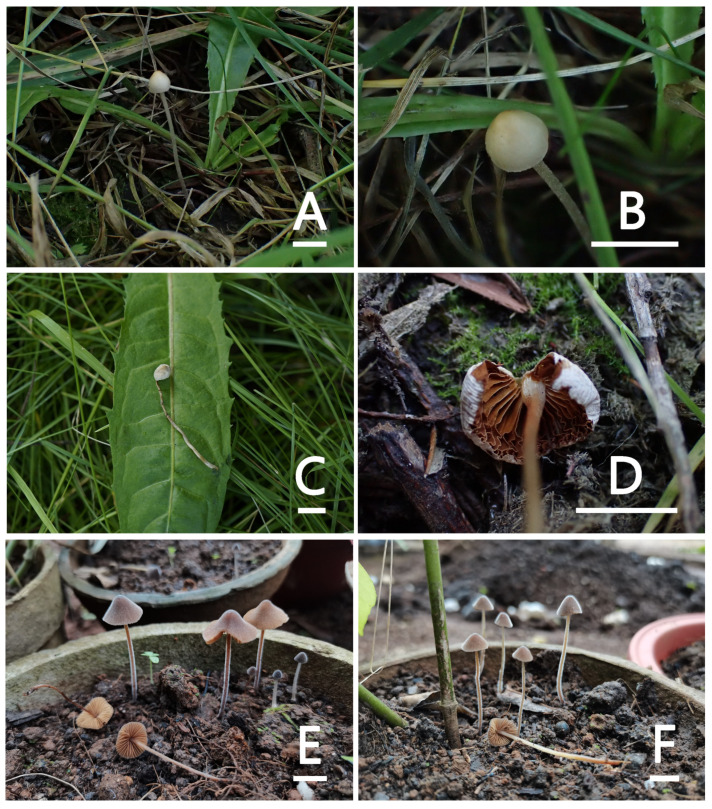

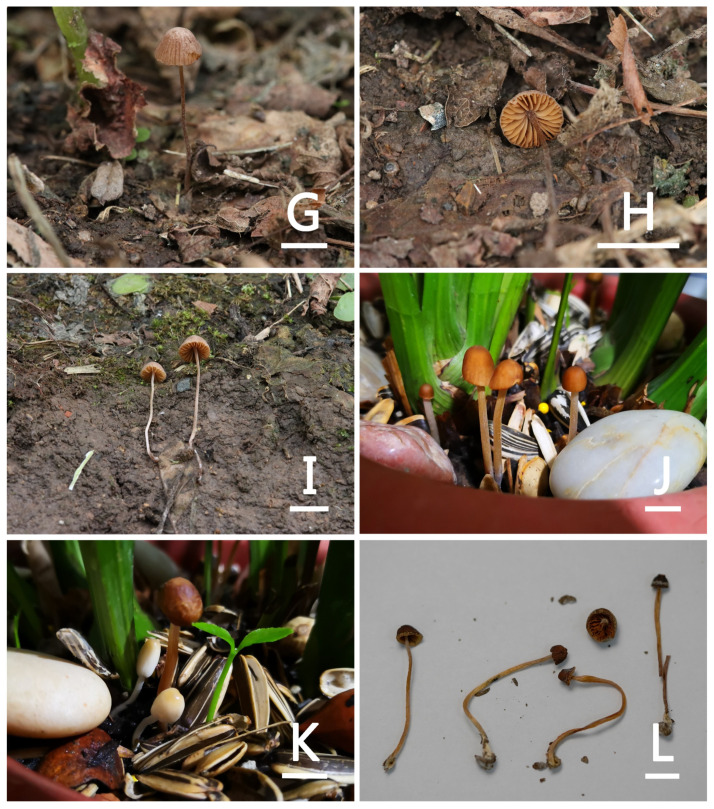

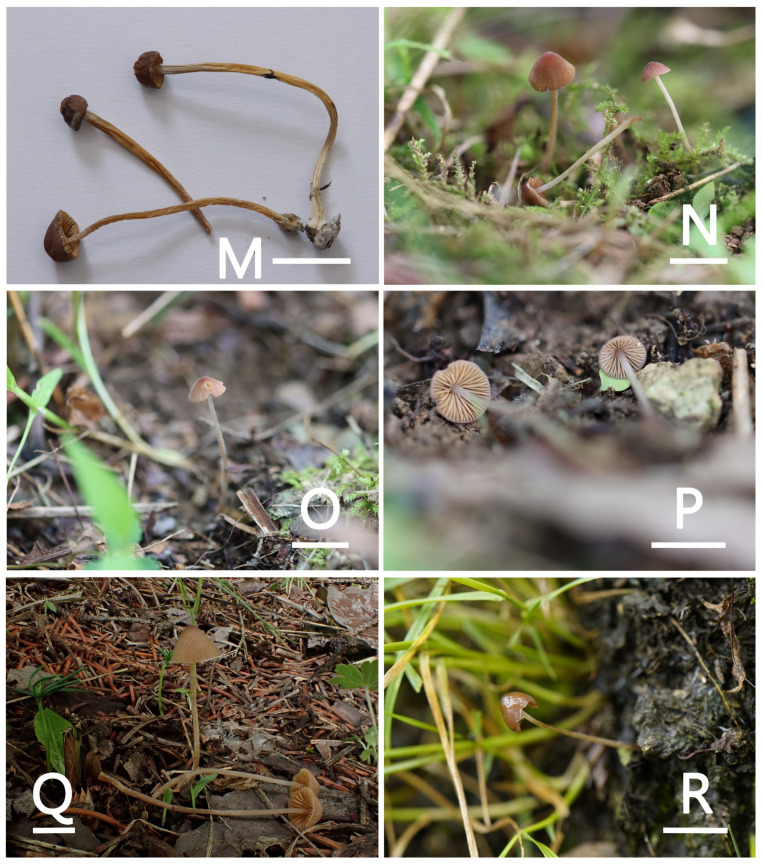

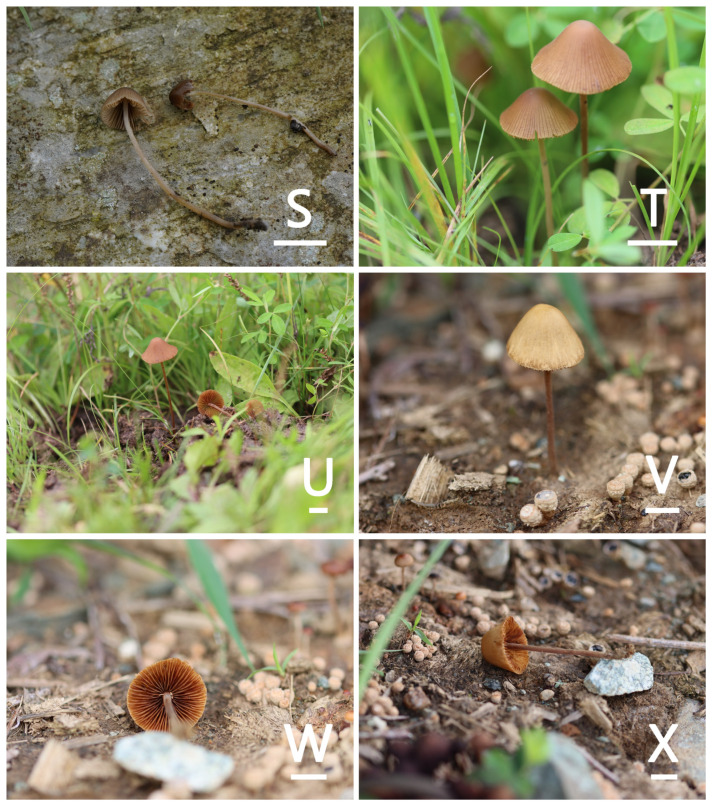
Basidiomata of Conocybe sect. Pilosellae species. (**A**–**D**) Conocybe pseudocrispa, (**E**,**F**) Conocybe pilosa, (**G**–**I**) Conocybe reniformis, (**J**–**M**) Conocybe ceracea, (**N**–**P**) Conocybe muscicola, (**Q**) Conocybe sinobispora, (**R**,**S**) Conocybe hydrophila, and (**T**–**X**) Conocybe rufostipes. Scale bars = 1 cm.

Specimens for this study were collected from different regions in China, including Tianjin Municipality, and the Inner Mongolia, Jilin, Heilongjiang, Gansu, Jiangsu, Hubei, Hunan, and Guangxi provinces. Field habitat photographs were taken (Figure 1), and specimens were deposited at the Mycology Herbarium of Jilin Agricultural University (HMJAU). The color of fresh basidiocarps was described using the color-coding system developed by the German Institute for Quality Assurance and Certification (Reichs-Ausschuss fur Lieferbedingungen und Guetesicherung, https://www.ral-guetezeichen.de/). The tissues of the specimens were treated with 5% KOH and 1% Congo red, and an ammonia reaction was carried out using a 25% ammonia solution [11]. Observations were made using a Carl Zeiss Primo Star optical microscope (Jena, Germany). The basidiospore measurements do not include the apiculus and are presented as ‘(a)b–c(d)’, where ‘b–c’ represents the minimum of 90% of the measured values and ‘a’ and ‘d’ represent the extreme values. The main body (sterigmata or excrescences not included) of the basidia, cheilocystidia, caulocystidia, and pileipellis were measured (if present). The notation (n/m/p) indicates that measurements were made on “n” randomly selected basidiospores from “m” basidiomes of “p” collections. Q is the ratio of length divided by width, and Qm represents the average quotient (length/width ratio) ± standard deviation.

### 2.2. DNA Extraction, PCR Amplification, and Sequencing

Genomic DNA was extracted from the dried specimens using a NuClean Plant Genomic DNA kit (ComWin Biotech, CW0531M, Taizhou, China) as per the manufacturer’s instructions. The primer pairs ITS1F/ITS4 [35], LR0R/LR7 [36], and EF1-983F/EF1-2212R [37] were used to amplify the ITS, nrLSU, and *tef1-α* sequences, respectively. The polymerase chain reaction (PCR) procedures were carried out according to the protocol described by Mou and Bau (2021) [38].

### 2.3. Phylogenetic Analyses

New sequences were uploaded to NCBI, while other sequences were downloaded from NCBI (Table 1). The ITS, nrLSU, and *tef1-α* sequences were aligned using the G-INS-i algorithm with two iterative cycles only, using the online Mafft tool (https://mafft.cbrc.jp/alignment/server/). The alignment was manually adjusted and trimmed using MEGA7, and the three introns in *tef1-α* were removed. In the multi-locus dataset (ITS + nrLSU + *tef1-α*) of *Conocybe*, 864 bp were measured for ITS, 1298 bp for nrLSU, and 927 bp for *tef1-α*. The concatenated alignment of ITS (1–864), nrLSU (865–2162), and *tef1-α* (2163–3089) comprised 102 sequences with 3089 columns, 1239 distinct patterns, 878 parsimony-informative sites, 258 singleton sites, and 1953 constant sites, and was generated using PhyloSuite [39]. The best-fit partition model (edge-unlinked) was selected using the BIC criterion with ModelFinder [40]. The best-fit model according to BIC was TIM2 + F + R4 for ITS and TIM2e + R4 for nrLSU + *tef1-α*. Maximum likelihood phylogenies were inferred using IQ-TREE under the edge-linked partition model for 5000 ultrafast bootstraps and the Shimodaira–Hasegawa-like approximate likelihood-ratio test [41,42]. The best-fit partition model (edge-unlinked) was again selected using the BIC criterion with ModelFinder, and the best-fit model according to BIC was GTR + F + I + G4 for ITS and SYM + I + G4 for nrLSU + *tef1-α*. Bayesian inference phylogenies were inferred using MrBayes 3.2.6 under the partition model with 2 parallel runs and 2,500,000 generations [43], with the initial 25% of sampled data discarded as burn-in. The final figures were edited using iTOL [44], Photoshop 2021 (Adobe), and Adobe Illustrator 2021 (Adobe).

## 3. Results

### 3.1. Molecular Phylogeny

**Figure 2:**
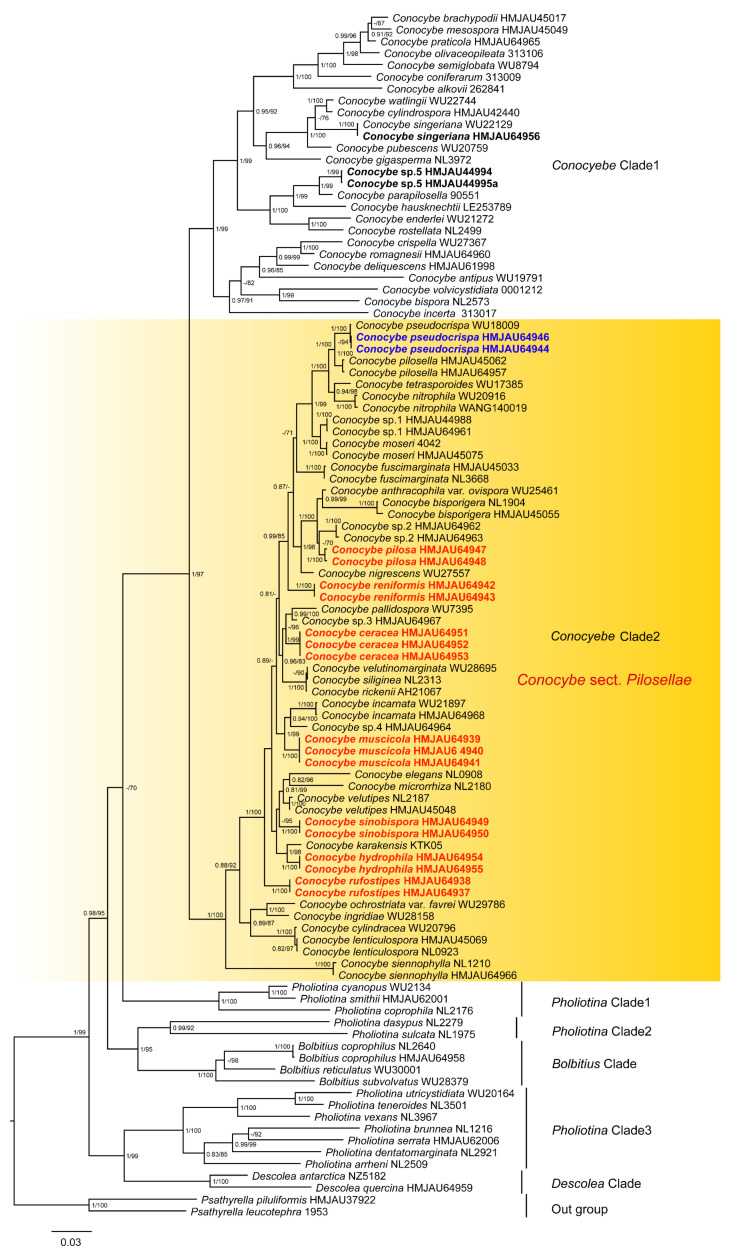
The phylogenetic relationships of *Conocybe* sect. *Pilosellae* in Bolbitiaceae using Bayesian inference and maximum likelihood methods based on a multi-locus dataset (ITS, nrLSU, and *tef1-α*). Nodes with PP (posterior probabilities) values ≥ 0.8 and ML bootstrap support values ≥ 70% are indicated in the phylogenetic tree. Sequences newly generated in this study are highlighted in colored font.

**Figure 3:**
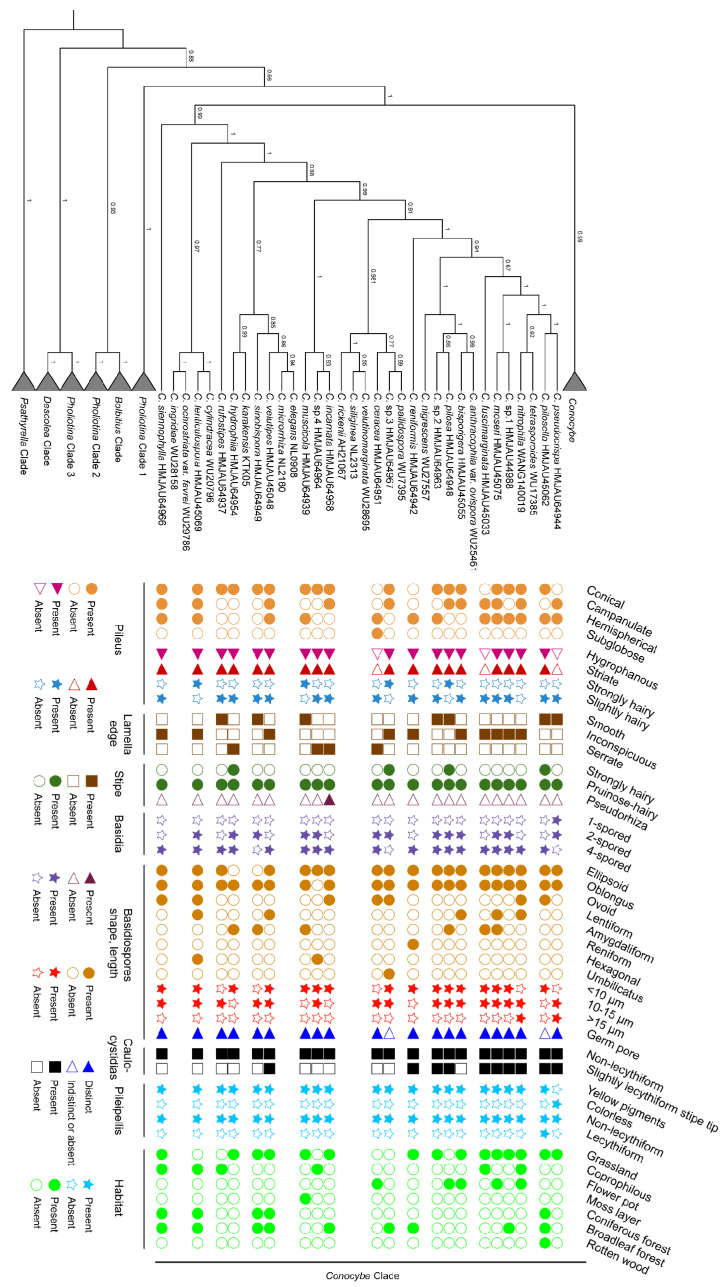
*Conocybe* sect. *Pilosellae* group was fitted with a morphology using a Bayesian phylogenetic tree based on a multi-locus dataset (ITS, nrLSU, tef1-α), and PP values are labeled on the nodes.

The Bayesian and ML phylogenetic trees were based on a combined dataset of ITS, nrLSU, and *tef1-α*. Only the Bayesian tree was presented due to the consistent topology between the Bayesian and ML trees. The support values were marked on the nodes of the tree. Only the data with Bayesian posterior probabilities (PP ≥ 0.8) and ML bootstrap values (MLbs ≥ 70%) were retained (Figure 2). In the phylogenetic tree of the Bolbitiaceae (Figure 2 and Figure 3), *Psathyrella* was used as the outgroup at the base of the tree. The tree showed that *Pholiotina* was a polyphyletic group, consisting of *Pholiotina* Clade1, *Pholiotina* Clade2, and *Pholiotina* Clade3. *Descolea*, *Bolbitius*, and *Conocybe* were monophyletic groups, and *Conocybe* was further divided into two clades, namely, *Conocybe* Clade1 and *Conocybe* Clade2. The morphological classification and phylogenetic analysis showed that *Conocybe* Clade2 belongs to the sect. *Pilosellae* and is located at the base of the *Conocybe* (Figure 2).

The bold black font on the molecular phylogenetic tree, *Conocybe* sp.5, was initially identified as *C. rostellata* (Velen.) Hauskn. & Svrček [20], belonging to the sect. *Pilosellae*, but its position in the phylogenetic tree was classified under *Conocybe* Clade1 (Figure 2). *C. singeriana* Hauskn. [56] was also located in *Conocybe* Clade1 on the phylogenetic tree (Figure 2).

The bold blue font in *Conocybe* Clade2 indicates that our specimens HMJAU64944 and HMJAU64946 cluster with *C. pseudocrispa* (Hauskn.) Arnolds WU18009 (1/100) on the phylogenetic tree. On the other hand, the bold red font indicates that our holotype HMJAU64947 (-/70) is a sister group to *Conocybe* sp.2. HMJAU64942 forms its own branch with a support value (0.99/85). HMJAU64951 (-/96) is a sister group to both *Conocybe* sp.3 and *C. pallidospora* Kühner & Watling. HMJAU64939 (1/99) is a sister group to both *Conocybe* sp.4 and *C. incarnata* (Jul. Schäff.) Hauskn. & Arnolds. HMJAU64949 (-/95) forms an independent branch. HMJAU64954 (1/98) is a sister group to *C. karakensis* T. Ullah & M. Saba. HMJAU64937 (1/100) forms its own independent branch.

Furthermore, as shown in Figure 3, by fitting morphological characteristics with phylogenetics, it has been found that the common features in the sect. *Pilosellae* are that all pilei have velvety hair and the caulocystidia are non-lecythiform. These two points confirm the origin of the naming of the sect. *Pilosellae* and its distinction from other sections, providing a basis for distinguishing various species within the sect. *Pilosellae* (for detailed inferences, refer to the Section 4).

### 3.2. Taxonomy

***Conocybe pilosa*** T. Bau & H. B. Song, **sp. nov.**

Mycobank No.: MB848242

**Figure 4:**
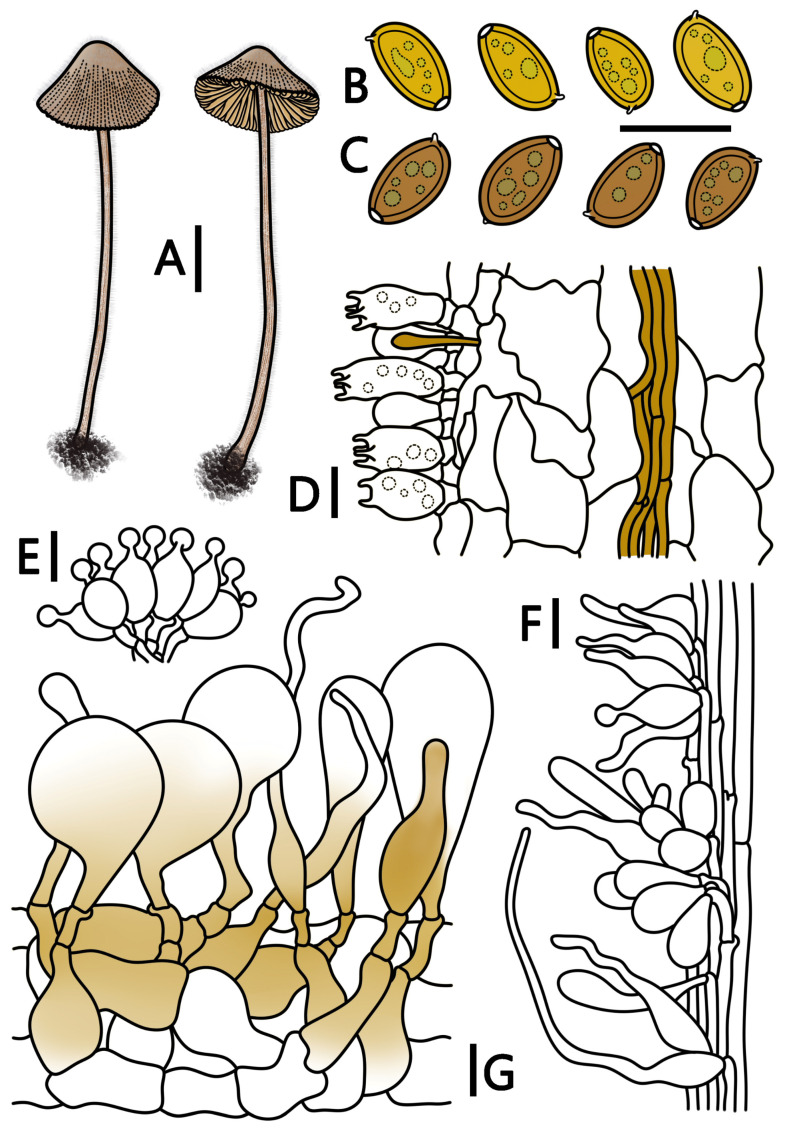
*Conocybe pilosa* (HMJAU 64947, HMJAU 64948) (**A**) basidiomata, (**B**) basidiospores in water, (**C**) basidiospores in KOH, (**D**) basidia, (**E**) cheilocystidia, (**F**) caulocystidia, and (**G**) pileipellis. (**A**) Scale bar = 1 cm; (**B**–**G**) scale bars = 10 μm.

Figure 1E,F and Figure 4

Etymology. “*pilosa*” refers to the dense hair on the pileus and stipe.

Holotypus. CHINA. Guangxi Zhuang Autonomous Region, Guilin City, Yanshan District, Guangxi Institute of Botany, 19 June 2022, 110°18’01″ E, 25°04’48″ N, alt. 170 m, Guangfu Mou, M22061902 (HMJAU 64947).

Diagnosis: The species has a blackish pileus, the surface of the pileus and stipe is covered with dense pubescence, the spores are slightly amygdaliform, four(two)-spored, and the pileipellis is composed of sphaeropedunculate and nearly clavate elements. Growing on potting soil.

Pileus diameter 0.5–2.0 cm, conical, campanulate, margin undulate, center ochre-yellow (RAL 1024), salmon pink (RAL 3012), and black-red (RAL 3007), edge ochre-yellow (RAL 1024) to terra brown (RAL 8003), hygrophanous, surface covered with dense pubescence, with distinct striate, can extend to the center of the pileus. Context thin, bright ivory (RAL 1015), beige (RAL 1001), without a distinctive odor. Lamellae adnate, moderately close, unequal, beige (RAL 1001), salmon pink (RAL 3012), smooth at the edge. Stipe 4.5–7.0 cm long, 1.0–2.0 mm thick, cylindrical, slightly enlarged at the base, bright ivory (RAL 1015), ochre-yellow (RAL 1024), covered with a powdery coating and pubescence, slightly longitudinally striated.

Spores (60/3/2) (8.2–)8.5–10.4(–11) × (5.1–)5.2–6.2(–6.8) μm, Q = 1.54–1.92, Qm = 1.68(±0.08), elliptical to elongate, slightly amygdaliform, wall slightly thick, containing oil droplets, germination pore diameter approximately 0.5–1.5 μm, spores signal yellow (RAL 1003) to lemon yellow (RAL 1012) in water, and honey yellow (RAL 1005) to ochre-brown (RAL 8001) in KOH. Basidia (13–)14–21(–22) × (7–)8–10(–12) μm, nearly elliptical, clavate, four(two)-spored, sterigmata 2–4 μm long, basidia with vacuolar contents. Cheilocystidia lecythiform, (13–)14–19(–20) × 6–11(–12) μm, with 3–5 µm wide capitula. Pleurocystidia chrysocystidia in KOH, cylindrical to clavate, and have yellow pigments. Caulocystidia nearly spherical, elliptical, cylindrical, clavate, lageniform, or lanceolate, up to 45 μm long, and occasionally also have hair-like caulocystidia up to 80 μm long, occasionally lecythiform near the stipe apex. Pileipellis hymeniform, consisting of sphaeropedunculate elements and nearly clavate elements, (32–)34–59(–60) × (18–)19–28(–36) μm, with yellow pigments at the base. Pileocystidia cylindrical, clavate, nearly lageniform, lanceolate, or hair-like, some with yellow pigments, up to 60 μm long. All tissues have clamp connections. Ammoniacal reaction is negative.

Habitat. Summer-growing, in potting soil.

Distribution. Currently, only known in the Guangxi Zhuang Autonomous Region, China.

Additional specimens measured. CHINA. Guangxi Zhuang Autonomous Region, Guilin City, Yanshan District, Guangxi Institute of Botany, 19 June 2022, 110°18’01″ E, 25°04’48″ N, alt. 170 m, Guangfu Mou, M22061903 (HMJAU 64948).

Notes. *C. pilosa* has a stipe that is covered with a powdery coating and pubescence, with slight longitudinal striations, similar to the stipe of *C. pilosella* (Pers.) Kühner, and spores of similar size. However, the pileus of *C. pilosella* has a brownish hue, and is four-spored, which distinguishes it from *C. pilosa*. The stipe of *C. pilosa* is also covered with a powdery coating and pubescence, with slight longitudinal striations, similar to the stipe of *C. pallidospora* Kühner & Watling, and is four(two)-spored with a similar pileipellis. However, the spores of *C. pallidospora* are very thin-walled, with small, often hardly visible germ pores, and there are no pileocystidia on the pileipellis, which distinguishes it from *C. pilosa*. The most closely related species of *C. pilosa* is *C. anthracophila* var. *ovispora* Hauskn., but the latter has a pileus that can reach up to 4.0 cm, with a brownish hue, four spores, and cheilocystidia up to 25 μm, making it easy to distinguish from *C. pilosa* [11,18].

***Conocybe reniformis*** T. Bau & H. B. Song, **sp. nov.**

Mycobank No.: MB848243

**Figure 5:**
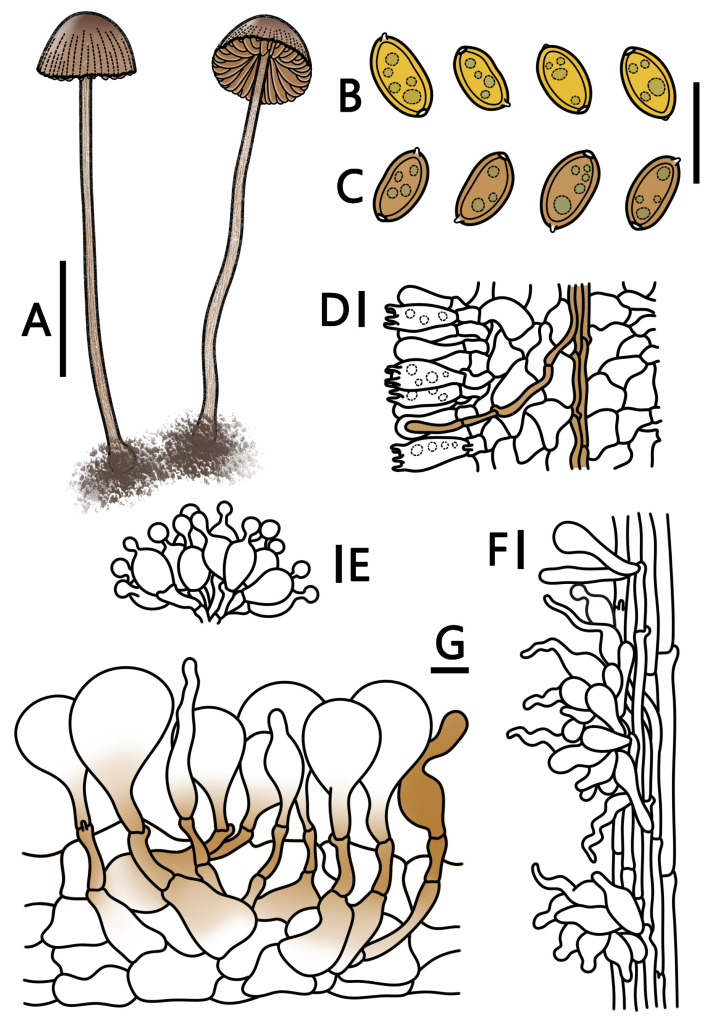
*Conocybe reniformis* (HMJAU 64942, HMJAU 64943) (**A**) basidiomata, (**B**) basidiospores in water, (**C**) basidiospores in KOH, (**D**) basidia, (**E**) cheilocystidia, (**F**) caulocystidia, and (**G**) pileipellis. (**A**) Scale bar = 1 cm; (**B**–**G**) scale bars = 10 μm.

Figure 1G–I and Figure 5

Etymology. “*reniformis*” refers to reniform spores.

Holotypus. CHINA. Jilin Province, Changchun City, Jingyuetan National Forest Park, 18 August 2022, 125°26’58″ E, 43°47’36″ N, alt. 225 m, Hanbing Song, S22081811 (HMJAU 64942).

Diagnosis: Basidioma small-sized, with the pileus center beige and the edge beige-brown (RAL 1011). The pileus of *C. reniformis* is hemispherical to paraboloid, with four-spored basidia and spores that are less than 10 μm in length and reniform. At the stipe apex, sporadically, there is also a single lecythiform caulocystidia similar to the cheilocystidia present. The pileocystidia are cylindrical and lageniform, some with yellow pigments, and show a weak positive reaction to ammonia. They grow on the ground in the grassland under the bushes of *Dasiphora*.

Pileus 0.5–1.0 cm wide, hemispherical to paraboloid, margin undulate, center bright ivory (RAL 1015) to beige (RAL 1001), and the edge beige brown (RAL 1011) to ochre-brown (RAL 8001), hygrophanous; surface smooth, not or faintly pubescent, with distinct striae, reaching up to 2/3 of the distance to the center. Context thin, the same color as the pileus, no special smell. Lamellae adnate, slightly thinner, unequal, beige (RAL 1001) to beige brown (RAL 1011), lamellar edge inconspicuous. Stipe 3.5–5.0 cm long, 1.0–2.0 mm thick, cylindrical, slightly enlarged at the base, forming a weak bulbous shape, the upper end of the stipe bright ivory (RAL 1015) to copper brown (RAL 8004), and the lower part is copper brown (RAL 8004) to reddish-brown (RAL 8012); surface covered with fine short hairs, slightly striated longitudinally.

Spores (60/3/2) (7.0–)7.5–9.2(–9.3) × (4.0–)4.4–5.2(–5.4) μm, Q = 1.53–1.86, Qm = 1.71(±0.07), elliptical to oblong, reniform, with slightly thick walls, containing oil droplets, and the germination pore diameter is less than 1 μm; spores signal yellow (RAL 1003) to lemon yellow (RAL 1012) in water, and honey yellow (RAL 1005) to ochre-brown (RAL 8001) in KOH. Basidia (15–)16–24(–25) × (7–)8–11 μm, clavate, four-spored, sterigmata 2–5 μm long, with vacuolar contents. Cheilocystidia lecythiform, 16–23(–25) × (7–)8–11(–12) μm, with 3–6 µm wide capitula. Pleurocystidia chrysocystidia in KOH, cylindrical to clavate, and have yellow pigments. Caulocystidia subglobose, lageniform, cylindrical, clavate, lanceolate, up to 40 μm long, and occasionally lecythiform near the stipe apex. Pileipellis hymeniform consisting of sphaeropedunculate elements, (24–)25–49(–55) × (14–)17–29(–30) μm, and yellow pigments at the base. Pileocystidia cylindrical, lageniform, some with yellow pigments, and can reach a length of 40 µm. All tissues have clamp connections. Weak positive reaction with ammonia water, forming diamond-shaped crystals.

Habitat. During the summer, they grow singly or scattered in the grassland under the bushes of *Dasiphora*.

Distribution. Currently, only known in Jilin Province, China.

Additional specimens measured. CHINA. Jilin Province, Changchun City, Jingyuetan National Forest Park, 18 August 2022, 125°26’58″ E, 43°47’36″ N, alt. 225 m, Hanbing Song, S22081812 (HMJAU 64943).

Notes. *C. reniformis* has a pileus color and spore size that are similar to those of *C. pilosella*. However, *C. pilosella* spores are ellipsoid-ovoid in shape, lacking a germ pore or with callus, and having only a few lecythiform pileocystidia, making it distinguishable from *C. reniformis*. *C. reniformis* has a pileus shape resembling that of *C. velutipes* (Velen.) Hauskn. & Svrček, bearing four-spored basidia and a few lecythiform canlocystidia near the stipe apex. However, *C. velutipes* has larger spores and lecythiform pileocystidia, making it distinguishable from *C. reniformis*. *C. reniformis* has a hemispherical pileus and four-spored basidia, with lecythiform canlocystidia occasionally present near the cheilocystidia at the stipe apex, similar to those of *C. nitrophila* (Hauskn.) Yen W. Wang & S.S. Tzean. Nonetheless, *C. nitrophila* spores are distinctly lentiform and longer than those of *C. reniformis*. Additionally, *C. nitrophila* has a negative ammoniacal reaction, making it distinguishable from *C. reniformis*. *C. reniformis* shares a similar basidioma morphology with *C. bispora* (Singer) Hauskn. and *C. ingridiae* Hauskn. However, the latter two species have two-spored basidia, making them distinguishable from *C. reniformis*. *C. reniformis* is closely related to *C. velutinomarginata* Hauskn. & Zugna, but the latter has an involute pileus and two-spored basidia, making it distinguishable from *C. reniformis* [11,18].

***Conocybe ceracea*** T. Bau & H. B. Song, **sp. nov.**

Mycobank No.: MB848245

**Figure 6:**
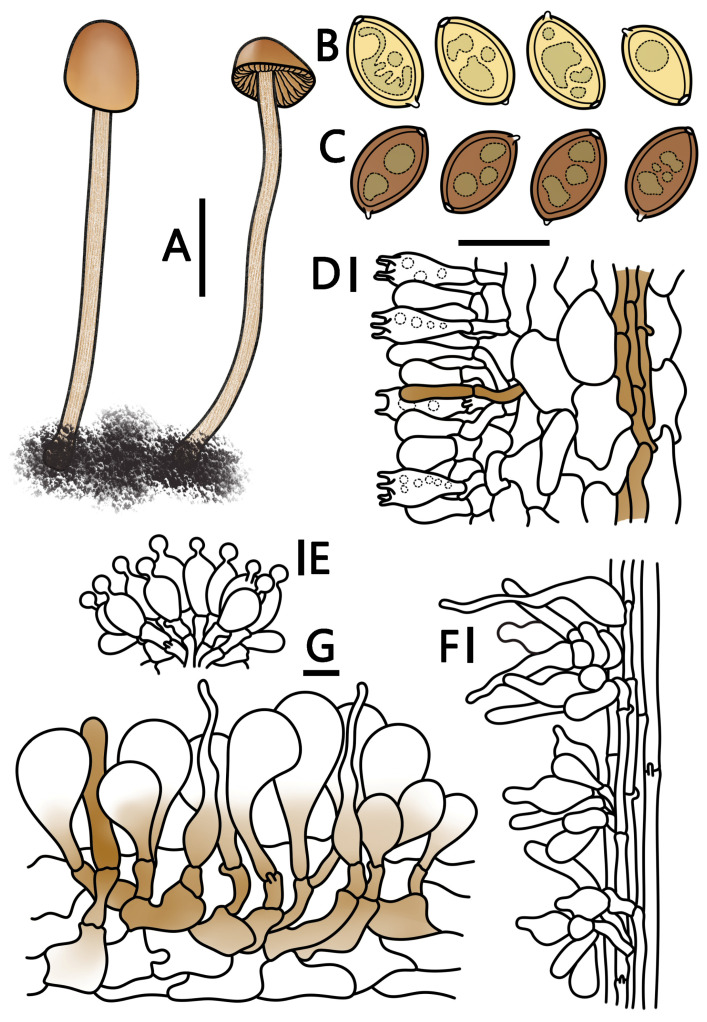
*Conocybe ceracea* (HMJAU 64951, HMJAU 64952) (**A**) basidiomata, (**B**) basidiospores in water, (**C**) basidiospores in KOH, (**D**) basidia, (**E**) cheilocystidia, (**F**) caulocystidia, and (**G**) pileipellis. (**A**) Scale bar = 1 cm; (**B**–**G**) scale bars = 10 μm.

Figure 1J–M and Figure 6

Etymology. “*ceracea*” here refers to the wax crystals that precipitate on the surface of the pileus after dehydration, as well as the waxy nature of the lamellae.

Holotypus. CHINA. Tianjin Municipality. Indoor potted plants in residential households. 22 February 2022, Liyang Zhu, TJ01 (HMJAU 64951).

Diagnosis: *C. ceracea* is often mistaken for *C. velutinomarginata* due to its subglobose pileus and brownish coloration, as well as its growth in potted plants. However, the surface of the pileus of *C. ceracea* is covered with inconspicuous short pubescence, is non-striate, and will exude yellowish-gray wax crystals after dehydration, which adhere to the surface of the pileus. The basidia are four(two)-spored and the spores are elliptical to ovoid, showing a weakly positive reaction to ammonia. It grows in the soil of potted Orchidaceae plants.

Pileus diameter 0.5–1.5 cm, subglobose, paraboloid to subcylindrical, margin incurved, moist surface dahlia yellow (RAL 1033) to ochre-brown (RAL 8001), dry surface clay brown (RAL 8003) to chestnut brown (RAL 8015); moist surface covered with inconspicuous short pubescence, without striate, after dehydration, yellowish-grey (RAL 7034) to ivory (RAL 1014); wax crystals precipitate on the surface of the pileus, mostly along the edge, forming a crusted lump. Context thin, concolorous to pileus, smell indistinct. Lamellae adnate, moderately close, unequal, ochre-brown (RAL 8001) to orange-brown (RAL 8023), with a whitish crenulate lamellar edge. Stipe 3.5–7.0 cm long, 1.0–2.0 mm thick, cylindrical, slightly enlarged at the base forming a bulb, honey yellow (RAL 1005) to melon yellow (RAL 1028), surface covered with a white powdery coating and fine pubescence, and slightly longitudinally striated.

Spores (60/3/3) (9.6–)10.2–11.7(–12.2) × (6.3–)6.5–7.5(–7.7) μm, Q = 1.38–1.79, Qm = 1.58(±0.07), elliptical to oblong, ovoid, containing oil droplets, germ pore diameter less than 1 μm, and sometimes indistinct, spores signal yellow (RAL 1003) to lemon yellow (RAL 1012) in water, and honey yellow (RAL 1005) to ochre-brown (RAL 8001) in KOH. Basidia (18–)19–27(–28) × 6–12(–13) μm, clavate, four(two)-spored, sterigmata 2–6 μm long, with vacuolar contents. Cheilocystidia lecythiform, (14–)15–23(–25) × (6–)7–11(–12) μm, with 3–5 um wide capitula. Pleurocystidia chrysocystidia in KOH, cylindrical to clavate, and have yellow pigments, lower than the hymenium. Caulocystidia subglobose, lageniform, cylindrical, or lanceolate, up to 55 μm long. Pileipellis hymeniform, consisting of sphaeropedunculate elements, (27–)31–60(–63) × (14–)15–30(–31) μm, with yellow pigments at the base. Pileocystidia cylindrical, lageniform, or lanceolate, some with yellow pigments, up to 60 μm long. All tissues have clamp connections. Weakly positive reaction with ammonia, forming diamond-shaped crystals.

Habitat. Growing solitarily or gregariously on the soil of potted Orchidaceae plants.

Distribution. Currently, only known in Tianjin Municipality, China.

Additional specimens measured. CHINA. Tianjin Municipality. Indoor potted plants in residential households. 18 February 2022, Liyang Zhu, TJ04 (HMJAU 64952), TJ05 (HMJAU 64953).

Notes. *C. ceracea* is similar to *C. velutinomarginata* in morphology and phylogeny. However, the wax crystals formed on the pileus of the former when dry may be mistaken for hairs, and the lamellae of the former are also waxy when dry. The spores of the latter are 11–16.5 × 7–10.5 μm, with a large germ pore, the basidia are two-spored, and the margin of the pileus has slender clavate elements [11,18].

***Conocybe muscicola*** T. Bau & H. B. Song, **sp. nov.**

Mycobank No.: MB848246

**Figure 7:**
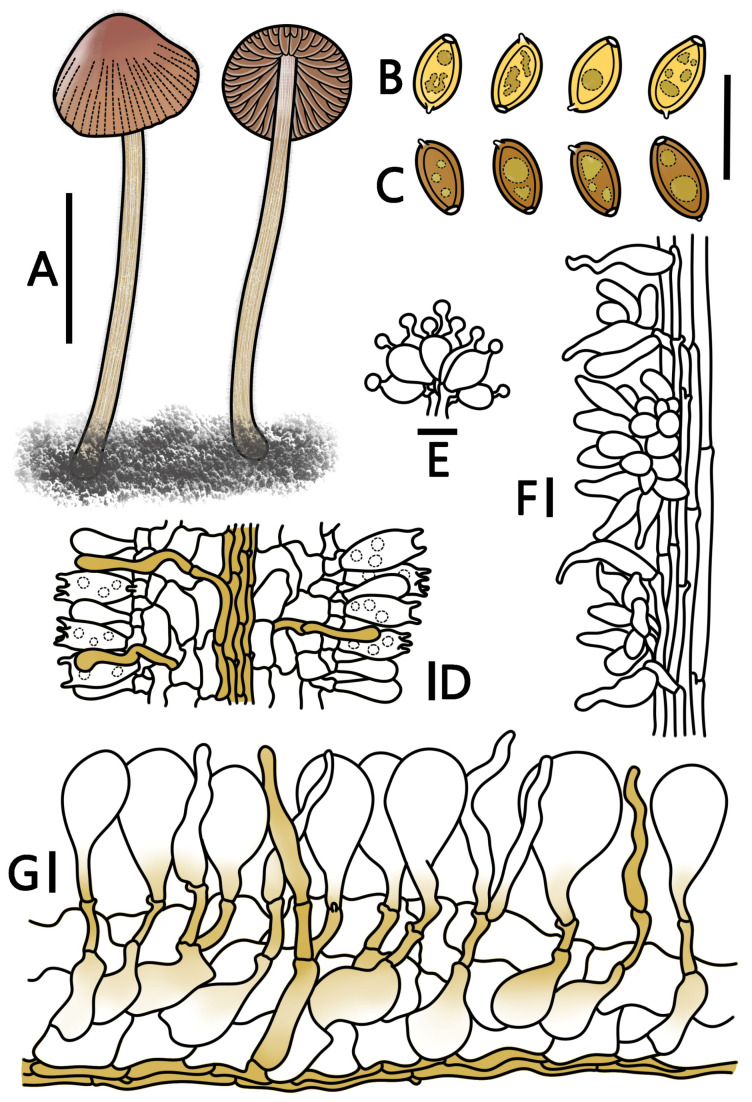
*Conocybe muscicola* (HMJAU 64939, HMJAU 64940) (**A**) basidiomata, (**B**) basidiospores in water, (**C**) basidiospores in KOH, (**D**) basidia, (**E**) cheilocystidia, (**F**) caulocystidia, and (**G**) pileipellis. (**A**) Scale bar = 1 cm; (**B**–**G**) scale bars = 10 μm.

Figure 1N–P and Figure 7

Etymology. “*muscicola*” refers to growing on grassy mossy layers.

Holotypus. CHINA. Hubei Province, Enshi Tujia and Miao Autonomous Prefecture, Shennongjia National Nature Reserve, 26 June 2022, 31°27’39″ E, 110°23’47″ N, alt. 1183 m, Hanbing Song, S22062603 (HMJAU 64939).

Diagnosis: Pileus surface of *C. muscicola* is hairy, center pearl orange (RAL 2013) to oxide red (RAL 3009). Pileus, four(two)-spored, with non-lecythiform caulocystidia. The cheilocystidia are relatively small, while the pileocystidia are cylindrical or lageniform, some with yellow pigments. Additionally, *C. muscicola* shows a weakly positive reaction to ammonia. Grows on grassy mossy layers in summer.

Pileus diameter 0.5–1.0 cm, conical, center pearl orange (RAL 2013) to oxide red (RAL 3009), margin beige-red (RAL 3012) to ivory (RAL 1014), hygrophanous; surface hairy, with distinct striate, reaching up to 2/3 of the distance to the center. Context thin, beige (RAL 1001) to sandy yellow (RAL 1002), with no distinctive odor. Lamellae adnate, slightly crowded, unequal, sandy yellow (RAL 1002) to beige-brown (RAL 1011), with smooth edges. Stipe 1.5–4.0 cm long, 1.0–2.0 mm thick, cylindrical, slightly enlarged at the base forming a weak bulbous shape, oyster white (RAL 1013), beige-brown (RAL 1011) to ochre-brown (RAL 8001), covered with powdery coating and fine hairs, slightly striated longitudinally.

Spores (60/3/3) (6.5–)7.0–9.0(–9.3) × (4.2–)4.4–5.5(–6.0) μm, Q = 1.44–1.87, Qm = 1.58(±0.09), elliptical to elongated elliptical, amygdaliform, with slightly thick walls and containing oil droplets, germination pore about 1 μm in diameter, spores signal yellow (RAL 1003) to lemon yellow (RAL 1012) in water, and honey yellow (RAL 1005) to ochre-brown (RAL 8001) in KOH. Basidia 13–21(–23) × 6–10(–11) μm, broadly clavate to clavate, with four(two)-spored, sterigmata 2–5 μm long, with vacuolar contents. Cheilocystidia are lecythiform, 13–20(–21) × 6–10 μm, with 3–5 µm wide capitula. Pleurocystidia chrysocystidia in KOH, cylindrical to clavate, and have yellow pigments. Caulocystidia subglobose, lageniform, cylindrical, clavate, lanceolate or with hairs; non-hairy cystidia up to 35 μm long. Pileipellis hymeniform consisting of spheropedunculate elements, 21–45(–50) × (12–)13–23(–24) µm, and yellow pigments at the base. Pileocystidia are cylindrical, lageniform, some with yellow pigments, and can reach a length of 40 µm. All tissues with clamp connections. Weakly positive with ammonia, forming diamond-shaped crystals.

Habitat. Solitary or scattered on grassy mossy layers in summer.

Distribution. Currently, only known in Hubei Province, China.

Additional specimens measured. CHINA. Hubei Province, Enshi Tujia and Miao Autonomous Prefecture, Shennongjia National Nature Reserve, 26 June 2022, 31°27’39″ E, 110°23’47″ N, alt. 1183 m, Hanbing Song, S22062604 (HMJAU 64940); 26 June 2022, 31°27’19″ E, 110°23’40″ N, alt. 1210 m, Hanbing Song, S22062611 (HMJAU 64941).

Notes. *C. muscicola* has a red-colored pileus that is hairy, like *C. rostellata*, and has similar spore sizes. However, *C. rostellata* has four-spored basidia with lecythiform caulocystidia, which are particularly found sporadically near the stipe apex, and a negative reaction to ammonia, distinguishing it from *C. muscicola*. *C. muscicola* and *C. ochrostriata* Hauskn. both have a red-colored pileus and almond-shaped spores. However, *C. ochrostraia* spores can reach up to 14 μm, and it has four-spored basidia with lecythiform caulocystidia, which are particularly found sporadically near the stipe apex. It also has clamp connections present, but rare, making it easy to distinguish from *C. muscicola*. *C. muscicola* has similar spore sizes and four(two)-spored basidia as *C. nigrescens* Hauskn. & Gubitz. However, the pileus of *C. nigrescens* is dark brown to black-brown with a yellow-brownish margin, and when fresh, it is non-striate. It also has a negative reaction to ammonia, distinguishing it from *C. muscicola*. *C. muscicola* and *C. microrrhiza* Hauskn. both have a red-colored pileus and lack lecythiform caulocystidia. However, the basidia of *C. microrrhiza* are two-spored, and it has lecythiform pileocystidia, which distinguishes it from *C. muscicola*. The closely related species to *C. muscicola* is *C. incarnata*, which also has a red-colored pileus and similar spore size. However, the pileus surface of *C. incarnata* is usually smooth, and may or may not be hairy, with the striae gradually disappearing, making it easy to distinguish from *C. muscicola* [11,18].

***Conocybe sinobispora*** T. Bau & H. B. Song, **sp. nov.**

Mycobank No.: MB848247

**Figure 8:**
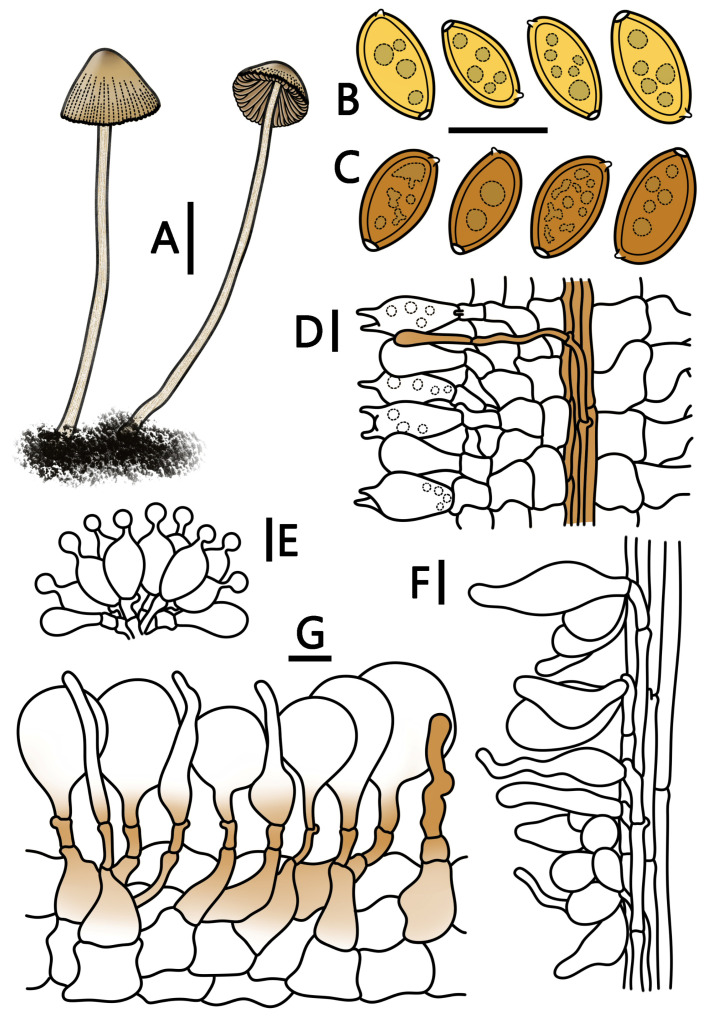
*Conocybe sinobispora* (HMJAU 64949) (**A**) basidiomata, (**B**) basidiospores in water, (**C**) basidiospores in KOH, (**D**) basidia, (**E**) cheilocystidia, (**F**) caulocystidia, and (**G**) pileipellis. (**A**) Scale bar = 1 cm; (**B**–**G**) scale bars = 10 μm.

Figure 1Q and Figure 8

Etymology. “*sinobispora*” refers to “*bispora*” from China.

Holotypus. CHINA. Jilin Province, Changchun City, Jilin Agricultural University Campus, 3 July 2022, 125°24’24″ E, 43°48’26″ N, alt. 293 m, Tolgor Bau, T22070309 (HMJAU 64949).

Diagnosis: *C. sinobispora* is often mistaken for *C. bispora*, as both have an ochre-yellow pileus that is smooth and slightly micaceous, with two-spored basidia and spores of similar size. However, the pileus of the former is conical, and its stipe is olive-yellow to ochre-yellow, with amygdaliform spores and cylindrical, lageniform, or hair-like pileocystidia. Additionally, the ammoniacal reaction of *C. sinobispora* is weakly positive. Growing on the ground in mixed forests.

Pileus diameter 0.5–1.5 cm, conical, margin undulate, center pearl gold (RAL 1036) to green-brown (RAL 8000), edge beige-brown (RAL 1011) to ochre-yellow (RAL 1024), hygrophanous, covered with short pubescence, and has distinct striate that can extend to 3/4 of the pileus radius. Context thin, and the same color as the pileus, without a distinctive odor. Lamellae adnate, moderately close, unequal, brown-beige (RAL 1011) to ochre-yellow (RAL 1024), with smooth edges. Stipe 3.0–4.0 cm long, 0.5–1.5 mm thick, cylindrical, slightly enlarged at the base forming a bulb, olive-yellow (RAL 1020) to ochre-yellow (RAL 1024); surface covered with a powdery coating and fine pubescence, and slightly longitudinally striated.

Spores (60/3/2) (10.6–)10.8–13.0(–13.5) × (5.9–)6.2–7.5(–7.7) μm, Q = 1.63–1.90, Qm = 1.75(±0.06), elongate-ellipsoid to amygdaliform, wall slightly thick, containing oil droplets, germination pore diameter approximately 1 μm, spores signal yellow (RAL 1003) to lemon yellow (RAL 1012) in water, and honey yellow (RAL 1005) to ochre-brown (RAL 8001) in KOH. Basidia (13–)14–21(–22) × (7–)8–10(–11) μm, broadly clavate to clavate, two-spored, sterigmata 2–6 μm long, with vacuolar contents. Cheilocystidia lecythiform, 14–21(–22) × 6–10(–11) μm, with 3–5 μm wide capitula. Pleurocystidia chrysocystidia in KOH, cylindrical to clavate, and have yellow pigments. Caulocystidia subglobose, lageniform, cylindrical, clavate, or lanceolate, up to 40 μm long. Pileipellis hymeniform, consisting of sphaeropedunculate elements, (21–)22–44(–46) × 16–31(–35) μm, with yellow pigments at the base. Pileocystidia cylindrical, lageniform, or hair-like, some with yellow pigments, up to 40 μm long. All tissues have clamp connections. Ammoniacal reaction is weakly positive, forming diamond-shaped crystals.

Habitat. Summer solitary or scattered on the ground in mixed forests.

Distribution. Currently, only known in Jilin Province, China.

Additional specimens measured. CHINA. Jilin Province, Changchun City, Jilin Agricultural University Campus, 3 July 2022, 125°24’24″ E, 43°48’26″ N, alt. 293 m, Siying Li, L22070310 (HMJAU 64950).

Notes. *C. sinobispora* is easily confused with the two-spored basidia species in *Conocybe* sect. *Pilosellae* (Figure 3). The difference between *C. sinobispora* and *C. bisporigera* (Hauskn. & Krisai) Arnolds is that the latter has a pileus with a blackish hue, distinctly lentiform spores, and a wider germ pore. The difference between *C. sinobispora* and *C. bispora* is that the latter has a pileus that is flat hemispherical to flat conical, with spores that are remarkably fusiform-limoniform in side-view and lacks pileocystidia. The difference between *C. sinobispora* and *C. umbellula* (Mont.) Singer is that the latter has spores that are remarkably cylindrical in face-view and elongate-limoniform in side-view. *C. microrrhiza* can be distinguished by its reddish-brown pileus and slightly lentiform spores. *C. inocybeoides* Watling has two(one)-spored basidia with larger basidiospores and cheilocystidia up to 30 μm, making it easy to distinguish. *C. velutinomarginata* has a regularly spherical pileus that later becomes slightly campanulate, non-striate, and has slender clavate elements on the pileus margin. *C. gigasperma* Enderle & Hauskn. has spores that can reach up to 25 μm. *C. siliginea* (Fr.) Kühner and *C. rickenii* (Jul. Schäff.) Kühner have larger basidiospores and two(one)-spored basidia [11,18].

***Conocybe hydrophila*** T. Bau & H. B. Song, **sp. nov.**

Mycobank No.: MB848248

**Figure 9:**
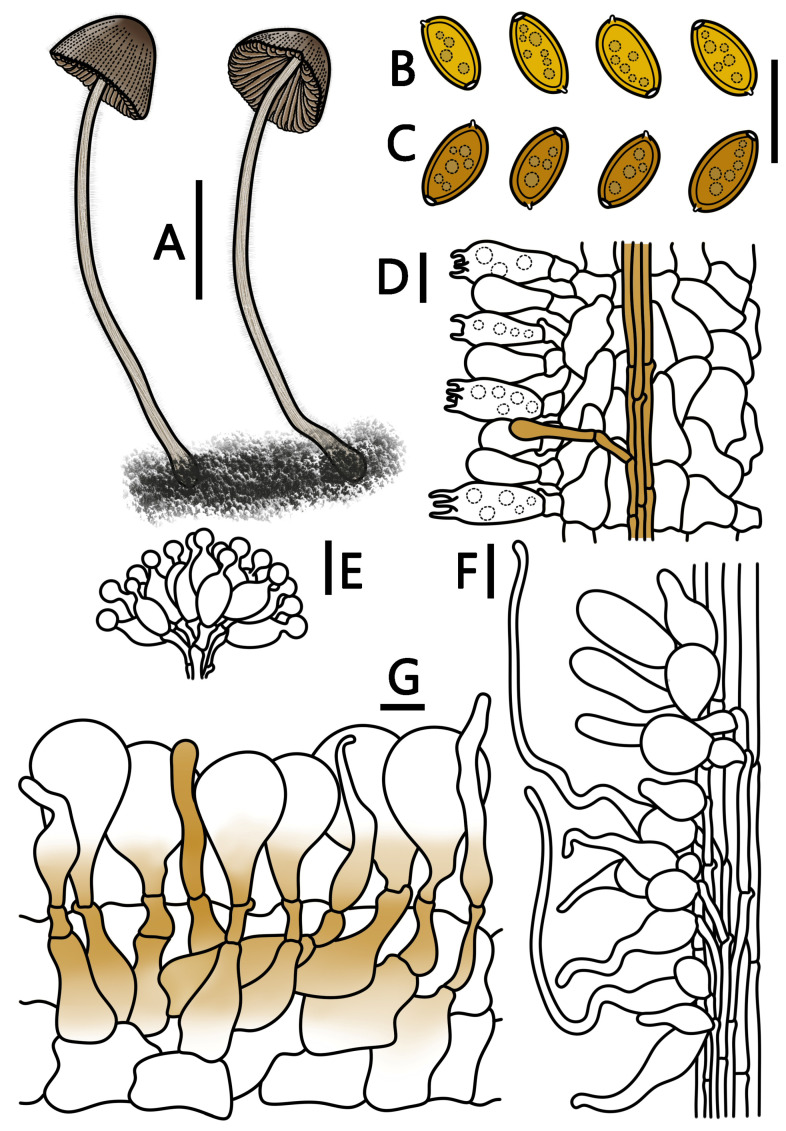
*Conocybe hydrophila* (HMJAU 64954) (**A**) basidiomata, (**B**) basidiospores in water, (**C**) basidiospores in KOH, (**D**) basidia, (**E**) cheilocystidia, (**F**) caulocystidia, and (**G**) pileipellis. (**A**) Scale bar = 1 cm; (**B**–**G**) scale bars = 10 μm.

Figure 1R,S and Figure 9

Etymology. “*hydrophila*” refers to an inclination or preference for moist conditions.

Holotypus. CHINA. Hunan Province, Shaoyang City, Chengbu Miao Autonomous County, Nanshan Pasture, 1 July 2022, 110°07’14″ E, 26°10’08″ N, alt. 1770 m, Liyang Zhu, Z22070112 (HMJAU 64954).

Diagnosis: *C. hydrophila* has small basidiomata, with a conical, nearly paraboloid pileus, the pileus hygrophanous, preferring moist conditions, lamellae serrate edge, and a slightly enlarged stipe base, but not bulbous. The spores are oblong to slightly amygdaliform, and the basidia are four(two)-spored. The caulocystidia non-lecythiform. grows in alpine meadows.

Pileus diameter 0.5–1.0 cm, conical, nearly paraboloid, center pearl gold (RAL 1036) to light terra brown (RAL 8028), edge ochre-yellow (RAL 1024) to khaki grey (RAL 7008), surface hygrophanous, non-viscid, covered with short pubescence, that gradually disappears, with distinct striate, up to 3/4 of the distance towards the center. Context thin, concolorous to pileus, without a distinctive odor. Lamellae adnate, moderately close, unequal, grey-beige (RAL 1019) to green-brown (RAL 8000), with a serrate edge. Stipe 3.0–4.5 cm long, 1.0–2.0 mm thick, cylindrical, slightly enlarged at the base, ochre-yellow (RAL 1024) to olive-brown (RAL 8008), surface covered with pubescence and slightly longitudinally striated.

Spores (40/2/2) (7.6–)7.7–9.5(–9.6) × (4.3–)4.4–5.3(–5.4) μm, Q = 1.61–1.93, Qm = 1.77(±0.07), oblong to slightly amygdaliform, containing oil droplets, with slightly thick wall and ca 0.5–1.0 um wide germ pore, spores signal yellow (RAL 1003) to lemon yellow (RAL 1012) in water, and honey yellow (RAL 1005) to ochre-brown (RAL 8001) in KOH. Basidia (12–)13–19(–20) × 6–9(–10) μm, broadly clavate to clavate, four(two)-spored, sterigmata 2–5 μm long, with vacuolar contents. Cheilocystidia are lecythiform, 12–18(–19) × 5–8(–9) μm, with 3–6 um wide capitula. Pleurocystidia chrysocystidia in KOH, cylindrical to clavate, and have yellow pigments. Caulocystidia subglobose, oblong, lageniform, long-necked lageniform, cylindrical, broadly clavate, or lanceolate, up to 40 μm long, and hair-like cystidia up to 100 μm long. Pileipellis hymeniform, consisting of sphaeropedunculate and clavate elements, (27–)28–47(–50) × (17–)18–30(–35) μm, with yellow pigments at the base. Pileocystidia cylindrical, broadly clavate, nearly lageniform, or hair, some with yellow pigments, up to 50 μm long. All tissues have clamp connections. Negative reaction with ammonia.

Habitat. Grows solitary in alpine meadows during the summer.

Distribution. Currently, only known in Hunan Province, China.

Additional specimens measured. CHINA. Hunan Province, Shaoyang City, Chengbu Miao Autonomous County, Nanshan Pasture, 30 June 2022, 110°07’18″ E, 26°10’22″ N, alt. 1655 m, Liyang Zhu, S22063010 (HMJAU 64955).

Notes. *C. hydrophila* has a hygrophanous pileus similar to *C. pallidospora*, with similar spore size and four(two)-spored basidia, but the latter has ellipsoid-pip-shaped spores with small, often hardly visible germ pore and lacks pileocystidia, which can be distinguished from *C. hydrophila*. It has a similar stipe base and spore size as *C. rostellata*, but the latter has a brownish pileus, four-spored basidia, and sporadically has single lecythiform caulocystidia at the stipe apex, which can be distinguished. It has a grayish-brown pileus and a slightly enlarged stipe base similar to *C. bisporigera*, but the latter has larger spores and two(one)-spored basidia, which can be distinguished. Phylogenetically, its closest relative is *C. karakensis*, which also has four(two)-spored basidia but has larger spores up to 15 μm, making it easy to distinguish [11,18].

***Conocybe rufostipes*** T. Bau & H. B. Song, **sp. nov.**

Mycobank No.: MB848249

**Figure 10:**
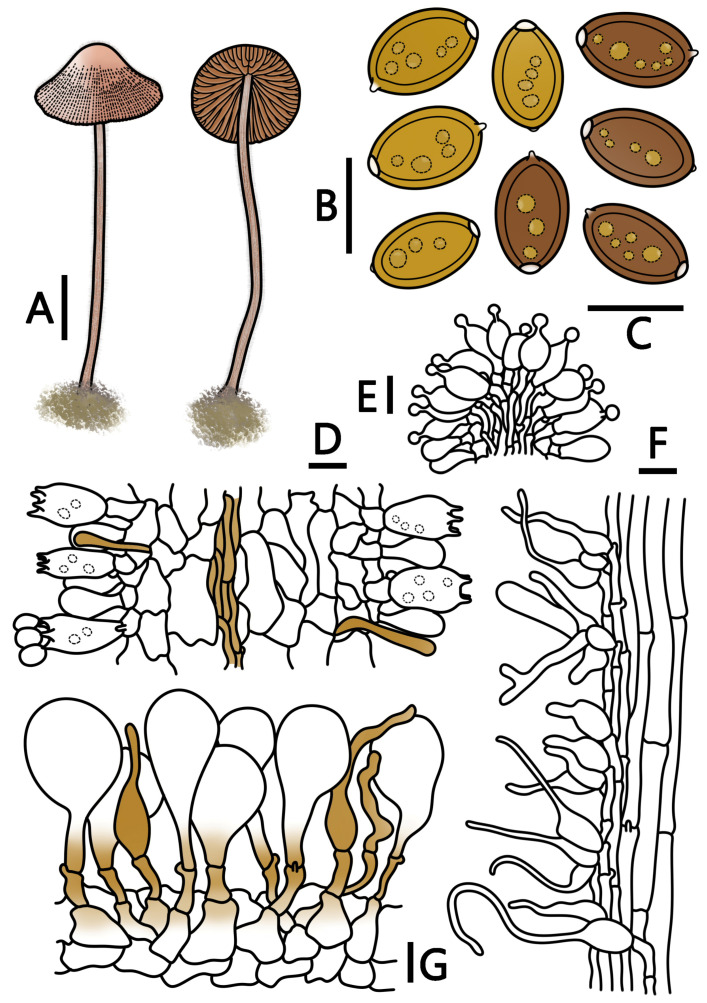
*Conocybe rufostipes* (HMJAU 64937, HMJAU 64938) (**A**) basidiomata, (**B**) basidiospores in water, (**C**) basidiospores in KOH, (**D**) basidia, (**E**) cheilocystidia, (**F**) caulocystidia, and (**G**) pileipellis. (**A**) Scale bar = 1 cm; (**B**–**G**) scale bars = 10 μm.

Figure 1T–X and Figure 10

Etymology. “*rufostipes*” refers to its’ reddish-brown stipe.

Holotypus. CHINA. Jilin Province, Siping City, Yitong Manchu Autonomous County, 19 July 2022, 125°12’19″ E, 43°37’28″ N, alt. 285 m, Hanbing Song, S22071903 (HMJAU 64937).

Diagnosis: *C. rufostipes* has wider spores and pileocystidia that are cylindrical to lanceolate in shape, and it shows a weakly positive reaction to ammonia, forming diamond-shaped crystals. Additionally, it has brown spots on the lamellae and reddish-brown stipe. Growing on cow dung.

Pileus 1.0–2.5 cm in diameter, conical with a broad convex center, and beige (RAL 1001) to sandy yellow (RAL 1002) in the center, edge ochre-brown (RAL 8001), orange-brown (RAL 8023), hygrophanous, surface smooth, faintly hairy, striations distinct, extending up to 4/5 of the radius from the edge to the center. Context thin, sandy yellow (RAL 1002), and odorless. Lamellae narrowly adnate, slightly crowded, unequal, ranging in color from maize yellow (RAL 1006) to ochre-brown (RAL 8001), margin smooth, surface spotted. Stipe 4.5–7.5 cm long, 2.0–3.0 mm thick, cylindrical, slightly swollen at the base, and copper brown (RAL 8004) to deer brown (RAL 8007), oxide red (RAL 3009), the surface is covered with short hairs and slightly longitudinally striped.

Spores (60/3/2) 11.0–13.5(–14.0) × (6.5–)7.0–8.5(–9.0) µm, Q = 1.40–1.70, Qm = 1.56(±0.07), elliptical to elongated, contains oil droplets, with thick walls and up to 1–2 µm wide germ pore, spores signal yellow (RAL 1003) to lemon yellow (RAL 1012) in water, and honey yellow (RAL 1005) to ochre-brown (RAL 8001) in KOH. Basidia (16–)17–26(–27) × (8–)9–13(–14) µm, broadly clavate to subcylindrical, four-spored, with a length of 2–5 µm. Cheilocystidia lecythiform, (13–)14–22(–24) × (5–)6–9 µm, with 2–4 µm wide capitula. Pleurocystidia chrysocystidia in KOH, cylindrical to clavate, with yellow pigment, and lower than the basidia. Stipitipellis mainly consists of subglobose, lageniform, cylindrical, clavate, and lanceolate, up to 50 µm in length. Pileipellis hymeniform consisting of spheropedunculate elements, (25–)26–46(–50) × 16–25 µm. Pileocystidia cylindrical, lageniform, and lanceolate, with yellow pigment and up to 50 µm long. All tissues have clamp connections. The reaction with ammonia is weakly positive, forming diamond-shaped crystals.

Habitat. Summer solitary or gregarious on cow dung in grasslands.

Distribution. Currently, only known in Jilin Province, China.

Additional specimens measured. CHINA. Jilin Province, Siping City, Yitong Manchu Autonomous County, 17 July 2022, 125°11’29″ E, 43°36’13″ N, alt. 368 m, Hanbing Song, S22071710 (HMJAU 64938).

Notes. *C. rufostipes* has non-lecythiform caulocystidia, which sets it apart from the species in *Conocybe* sect. *Pilosellae* that grow on dung and can be easily confused with it. The difference between *C. rufostipes* and *C. siennophylla* (Berk. & Broome) Singer ex Chiari & Papetti is that the latter has smaller spores than the former. The presence of lecythiform caulocystidia, especially sporadically near the stipe apex, distinguishes *C. rufostipes* from *C. anthracophila* Maire & Kühner ex Kühner & Watling. *C. leporina* (Velen.) Singer grows on or near rabbit dung, has larger spores, and has two spores. The presence of lecythiform caulocystidia, especially sporadically near the stipe apex, distinguishes *C. rufostipes* from *C. fuscimarginata* (Murrill) Singer. *C. rickenii* has two (one) spores. *C. fimetaria* Watling has lentiform spores. *C. viridibrunnescens* E. Ludw. grows on horse dung and has slightly lentiform spores. *C. murinacea* Watling grows on horse dung and has a slightly eccentric germ pore. *C. farinacea* Watling has larger spores. *C. magnispora* (Murrill) Singer has spores that can reach up to 20 μm. *C. brunneidisca* (Murrill) Hauskn. has angular-hexagonal or slightly compressed spores. In terms of phylogeny, *C. rufostipes* is closely related to *C. velutipes*, but the latter grows in deciduous and coniferous forests, as well as in grassy habitats, and has lentiform spores [11,18,47].

***Conocybe pseudocrispa*** (Hauskn.) Arnolds

Synonymy. *Conocybe albipes* var. *pseudocrispa* Hauskn., *Öst. Z. Pilzk.* 7: 106 (1998)

**Figure 11:**
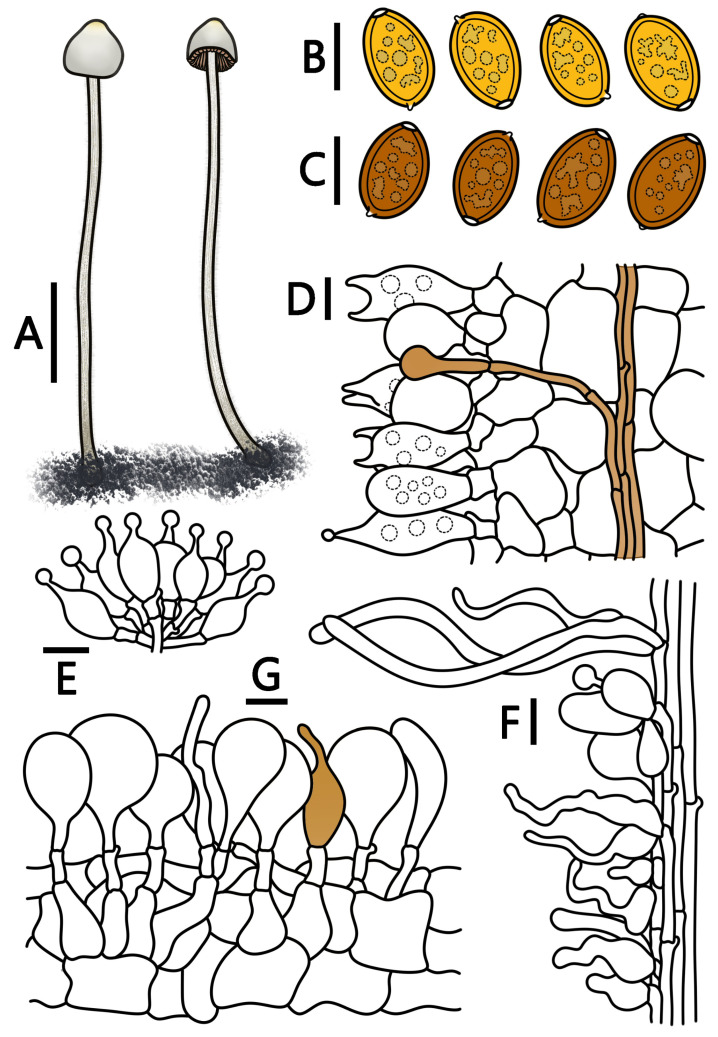
*Conocybe pseudocrispa* (HMJAU 64944, HMJAU 64945) (**A**) basidiomata, (**B**) basidiospores in water, (**C**) basidiospores in KOH, (**D**) basidia, (**E**) cheilocystidia, (**F**) caulocystidia, and (**G**) pileipellis. (**A**) Scale bar = 1 cm; (**B**–**G**) scale bars = 10 μm.

Figure 1A–D and Figure 11

Pileus 0.5–1.5 cm wide, hemispherical, campanulate to obtusely conical, center green-beige (RAL 1000) to ivory (RAL 1014), and beige (RAL 1001), in marginal zone often pure white (RAL 9010), oyster white (RAL 1013), surface smooth, not or faintly pubescent, non-striate, but old specimens have distinct striations. Context hyaline white, rather brittle, without smell and taste. Lamellae narrowly adnate, slightly ventricose, moderately distant, beige-brown (RAL 1011) to ochre-brown (RAL 8001), lamellae margin is smooth and not deliquescent. Stipe 3.0–5.0 cm, 1.0–1.5 mm thick, cylindrical with a gradually thickened base, stipe oyster white (RAL 1013), ochre-yellow (RAL 1024), covered with powdery and fine hairs, and slightly longitudinally striated.

Spores (60/3/3) (14.3–)14.5–16.8(–17.5) × (8.4–)8.7–10.8(–11.3) µm, Q = 1.45–1.80, Qm = 1.60(±0.10), ellipsoid to oblong, contains oil droplets, with thick walls and up to 1.5–2.5 µm wide germ pore, spores signal yellow (RAL 1003) to lemon yellow (RAL 1012) in water, and honey yellow (RAL 1005) to ochre-brown (RAL 8001) in KOH. Basidia 16–27(–28) × (8–)9–12(–13) µm, clavate, two(one)-spored, with a length of 4–8 µm, contain vacuolar content. Cheilocystidia lecythiform, (15–)16–21(–22) × (6–)7–11(–12) µm, with 3–5 µm wide capitula. Pleurocystidia chrysocystidia in KOH, cylindrical to clavate, and have yellow pigments. Pseudoparaphyses indistinct, clavate to subglobose. Stipitipellis mainly consists of subglobose, lageniform, cylindrical, clavate, lanceolate, up to 40 µm in length, hairs cystidia up to 100 µm, in between single lecythiform caulocystidia not rare. Pileipellis hymeniform, consisting of spheropedunculate and clavate elements, (20–)23–44(–46) × (14–)15–26(–30) µm, and lacks yellow pigments at the base. Pileocystidia are cylindrical, lageniform, and clavate, some of which have yellow pigments, and can reach a length of 40 µm. Clamp connections in all organizations. Ammoniacal reaction is negative.

Habitat. During the summer, it grows solitarily or scattered in grasslands under willow trees in riverbeds.

Distribution. Currently, only known in the Inner Mongolia Autonomous Region, China.

Additional collections were examined. CHINA. Inner Mongolia, Hulunbuir City, New Barag Left Banner, 7 August 2022, 119°27’23″ E, 47°27’22″ N, alt. 893 m, Hanbing Song, S22080707 (HMJAU 64944), Liyang Zhu, Z22080712 (HMJAU 64945), Shien Wang, E2208150 (HMJAU 64946).

Notes. *C. pseudocrispa* was previously classified in *Conocybe* sect. *Candidae* due to the presence of pseudoparaphyses, but as it is not deliquescent and the pseudoparaphyses are not distinct, its stipe morphology matches the characteristics of *Conocybe* sect. *Pilosellae*. Based on a phylogenetic tree constructed using ITS, 28S, and *tef1*-α, it has been proven that *C. pseudocrispa* belongs to *Conocybe* sect. *Pilosellae*. The main feature of *C. pseudocrispa* is its white pileus, and the pileipellis lacks yellow pigments at the base. Additionally, the pileocystidia are cylindrical, lageniform, clavate, and some of them have yellow pigments [11,18].

### 3.3. Confirmed Species of Conocybe Sect. Pilosellae in China

1. *Conocybe pilosella* (Pers.) Kühner [20];
2. *Conocybe pseudocrispa* (Hauskn.) Arnolds (this study);
3. *Conocybe nitrophila* (Hauskn.) Yen W. Wang & S.S. Tzean [49];
4. *Conocybe moseri* Watling [20];
5. *Conocybe fuscimarginata* (Murrill) Singer [20,47];
6. *Conocybe bisporigera* (Hauskn. & Krisai) Arnolds [20];
7. *Conocybe pilosa* T. Bau & H. B. Song (this study);
8. *Conocybe reniformis* T. Bau & H. B. Song (this study);
9. *Conocybe ceracea* T. Bau & H. B. Song (this study);
10. *Conocybe incarnata* (Jul. Schäff.) Hauskn. & Arnolds [29].

As recorded in Li and Azbukina (2011) [29]. Collection: Diaosui Lake, Songjiang Town, Jiaohe City, Jilin Province, China, 16 August 2021, HMJAU64968.

11. *Conocybe muscicola* T. Bau & H. B. Song (this study);
12. *Conocybe velutipes* (Velen.) Hauskn. & Svrček [20];
13. *Conocybe sinobispora* T. Bau & H. B. Song (this study);
14. *Conocybe hydrophila* T. Bau & H. B. Song (this study);
15. *Conocybe rufostipes* T. Bau & H. B. Song (this study);
16. *Conocybe lenticulospora* Watling [20];
17. *Conocybe siennophylla* (Berk. & Broome) Singer ex Chiari & Papetti.

As recorded in Li and Azbukina (2011) [29]. Collection: Jilin Agricultural University Campus, Changchun City, Jilin Province, China, 31 August 2022, HMJAU64966; Kezuohou Banner, Tongliao City, Inner Mongolia, China, 21 August 2022, HMJAU64969, HMJAU64970.

### 3.4. Hypothesized Species of Conocybe Sect. Pilosellae in China

1. *Conocybe* sp.1 (Figure 3)

Collection: Jilin Agricultural University Campus, Changchun City, Jilin Province, China, 3 July 2016, HMJAU44988, HMJAU64961.

2. *Conocybe* sp.2 (Figure 3)

Collection: The green belt of Xincheng Avenue, Changchun City, Jilin Province, China, 22 July 2022, HMJAU64962, HMJAU64963.

3. *Conocybe* sp.3 (Figure 3)

Collection: Purple Mountain, Nanjing City, Jiangsu Province, China, 30 April 2022, HMJAU64967.

4. *Conocybe* sp.4 (Figure 3)

Collection: Shansongling, Jiaohe City, Jilin Province, China, July 26, 2022, HMJAU64964.

### 3.5. Excluded and Doubtful Conocybe Sect. Pilosellae Species in China

1. *Conocybe rostellata* (Velen.) Hauskn. & Svrček [20]

Specimens HMJAU44994 and HMJAU44995a were re-examined and ITS was extracted. The species should be identified as *Conocybe* sp. and molecularly classified under *Conocybe* Clade1, and therefore should be excluded from the sect. *Pilosellae*. HMJAU44995 was re-identified as *C. velutipes*, and therefore it should be classified under sect. *Pilosellae* according to Liu (2018) [20].

2. *Conocybe singeriana* Hauskn. [11,18,56]

Collection: Mulun Township, Huanjiang Maonan Autonomous County, Hechi City, Guangxi Zhuang Autonomous Region, China. 9 April 2021, HMJAU64956. Although the caulocystidia morphology of *C. singeriana* belongs to the sect. *Pilosellae*, it is molecularly classified under *Conocybe* Clade1 and should be excluded from the sect. *Pilosellae*.

### 3.6. A Key to China Species of Conocybe Sect. Pilosellae


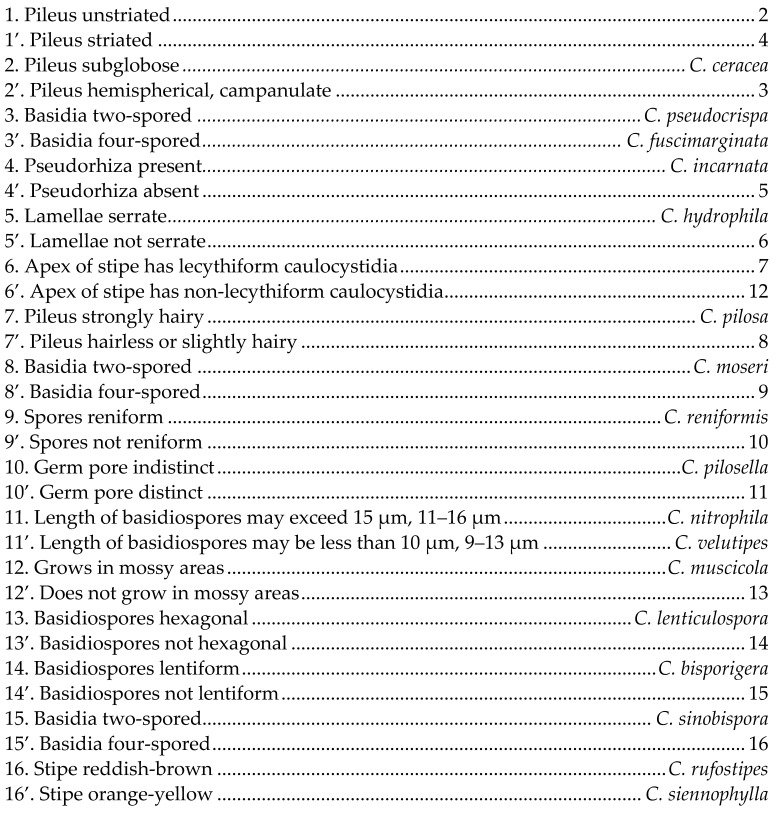


## 4. Discussion

Using the phylogenetic framework established by Tóth et al. (2013) and the morphological classification of Hausknecht (2009) [11,19], we incorporated genetic sequences from Chinese samples and combined the ITS, nrLSU, and tef1-α datasets to reconstruct the phylogenetic tree. The analysis revealed that *Conocybe* was a monophyletic group, whereas *Pholiotina* was a polyphyletic group. Moreover, the sect. *Pilosellae* was the basal section of *Conocybe*. This result has also been confirmed in the studies by Tóth et al. (2013) [19], Liu (2018) [20], and Ullah et al. (2023) [21]. *Pilosellae* is necessary because this section exhibits diverse forms of species evolution at the basal node of *Conocybe* (Figure 1 and Figure 3). For example, *C. pseudocrispa*, which has pseudoparaphyses, shares similarities with the species in the sect. *Candidae* and was previously classified in this section [11,57]. *C. incarnata* has pseudorhiza, *C. reniformis* has reniform spores, and *C. lenticulospora* Watling has hexagonal spores. The author believes that similar to convergent evolution, the evolutionary direction of the sect. *Pilosellae*, as a basal section, reflects the evolutionary characteristics of *Conocybe* [11]. Moreover, this section plays a crucial role in establishing the connection between *Pholiotina* and *Conocybe*, and provides a key clue for defining the boundaries between *Pholiotina* and *Conocybe* and resolving the issue of the polyphyletic origin of *Pholiotina*.

The Bayesian phylogenetic tree, based on the combined ITS, nrLSU, and tef1-α sequences (Figure 3) of the known species of the sect. *Pilosellae* from China, attempted to align morphological classification characteristics with molecular systematics to determine the classification and evolutionary characteristics of this section. It was discovered that the shape of the pileus of the species in this section is predominantly conical, with campanulate and hemispherical shapes being less common, and subglobose being the least common. A hygrophanous pileus may be correlated with the presence or absence of striations. Most species in this section have a slightly hairy pileus, with only a few species having a highly hairy pileus. The edges of the lamellae are either smooth or serrated. The stipes of all species in this section are hairy, with *C. incarnata* being the only species with pseudorhiza. Species with two-spored basidia were present on each of the smaller evolutionary branches. Although the spores also had other shapes, such as hexagonal, they were primarily ellipsoid to oblong in shape. Distinct germ pores have also been used to classify certain species. Whether the caulocystidia are lecythiform is the most important feature used to distinguish between different sections of the *Conocybe* [11]. At the end of the advanced evolutionary clade in this section, the species had a few lecythiform caulocystidia at the top of the stipe, indicating that the caulocystidia evolved to be lecythiform from non-lecythiform. If mapped to *Conocybe*, this represents an evolution from the sect. *Pilosellae* to sect. *Conocybe*, with the sect. *Mixtae* forming the transitional section [58]. The final-stage species of the sect. *Pilosellae* were frequently confused with species in the sect. *Mixtae*, which is why some species in the sect. *Pilosellae* have been categorized in the sect. *Mixtae* [11,58]. Pileocystidia are also a crucial feature of this section, even of the Bolbitiaceae. Pileocystidia were present in all the specimens observed; however, they are not easily observable in older specimens [11]. The evolutionary trend of pileocystidia was similar to that of caulocystidia, evolving from non-lecythiform to lecythiform. The evolutionary characteristics of species of this section are divergent, and features that would facilitate division into series have not yet been identified. However, this study provides crucial information on the subdivision of the sect. *Pilosellae* into different series.

Further phylogenetic analysis of the new species of the sect. *Pilosellae* (Figure 3) revealed that *C. rufostipes* mainly grew on cow dung with a brownish stipe, and formed a separate evolutionary branch on the phylogenetic tree. *C. hydrophila* formed the sister group of *C. karakensis* in the phylogenetic tree; however, *C. karakensis* grows in tropical sandy soils, whereas *C. hydrophila* grows in high mountain grasslands with different colors [21]. *C. sinobispora* can grow in coniferous forests and has two-spored basidia, unlike other species in this section, and it does not possess the second intron of *tef1-α* (983F/2212R). Within the clades *C. muscicola*, *Conocybe* sp.4, and *C. incarnata*, *C. muscicola* grows on moss layers, *Conocybe* sp.4 grows on cow dung, and *C. incarnata* grows on the ground in broad-leaved forests. Despite their different habitats, they all had reddish-hued pilei. Although the pilei of many species are hygrophanous, meaning that their color changes with the degree of moisture, the importance of color does not decrease. Further research is needed to determine whether this evolutionary branch can be established as a series. The pilei of *C. ceracea* and *C. velutinomarginata* have similar features; however, the waxiness of the *C. ceracea* pileus is distinct, thus being easily distinguishable from *C. velutinomarginata* [11]. A typical feature of *C. reniformis* is the presence of reniform spores, resulting in the formation of a separate evolutionary branch. Additionally, *C. pilosa* formed the sister group of *Conocybe* sp.2; however, it can be easily distinguished from the latter by its densely hairy pileus and stipe and smooth lamellar edges. The most distinctive feature of *C. pseudocrispa* is its white pileus with no pigment at the base of the pileipellis. However, the presence of pseudoparaphyses suggests that this is not the only sectional characteristic of the sect. *Candidae*; for instance, deliquescence is another sectional characteristic [11,57].

This article provides a comprehensive list of all the currently known species in the sect. *Pilosellae* from China and their corresponding sequences. This study attempted to integrate morphological classification with molecular systematics, but did not conduct a systematic study on *Conocybe* Clade1. Therefore, the sect. *Pilosellae* was not revised, and only some key information is provided, laying the foundation for future research. Currently, some species in the sect. *Pilosellae* have a few lecythiform caulocystidia at the top of the stipe, which can easily be misidentified as those in the sect. *Mixtae*. Meanwhile, some species possessing the characteristics of the sect. *Pilosellae* have been found in other sections [11,58]. To address these issues, a more comprehensive study of *Conocybe* species that integrates morphological and systematic approaches to elucidate the relationships between different sections is necessary.

## Figures and Tables

**Table 1:** Information on the DNA sequences used to reconstruct phylogenetic trees. Sequences newly generated in this study are indicated in bold.

Taxon	Voucher ID	ITS	nrLSU	*tef1-α*	Origin	References
***Bolbitius coprophilus***	**HMJAU64958**	**OQ780315**	**OQ758216**	–	**China**	**This study**
*B. coprophilus*	NL-2640	DQ234567	DQ234567	DQ234567	Hungary	[19]
*B. reticulatus*	WU30001	JX968249	JX968366	JX968455	Hungary	[19]
*B. subvolvatus*	WU28379	JX968248	JX968365	JX968454	Italy	[19]
*C. alkovii*	262841	JQ247196	–	–	Russia	[45]
*C. anthracophila* var. *ovispora*	WU25461	JX968237	JX968355	–	Italy	[19]
*C. antipus*	WU19791	JX968215	JX968332	JX968432	Austria	[19]
*C. bispora*	NL-2573	JX968203	JX968320	JX968423	Hungary	[19]
*C. bisporigera*	HMJAU45055	OP526418	–	–	China	[20]
*C. bisporigera*	NL-1904	JX968235	JX968353	JX968446	Hungary	[19]
*C. brachypodii*	HMJAU45017	MH141423	–	–	China	[20]
***C. ceracea***	**HMJAU64951**	**OQ758110**	**OQ758218**	**OQ758305**	**China**	**This study**
***C. ceracea***	**HMJAU64952**	**OQ758111**	**OQ758219**	**OQ758306**	**China**	**This study**
***C. ceracea***	**HMJAU64953**	**OQ758112**	**OQ758220**	–	**China**	**This study**
*C. coniferarum*	313009	NR_155030	–	–	Russia	[46]
*C. crispella*	WU27367	JX968208	JX968325	JX968426	Australia	[19]
*C. cylindracea*	WU20796	JX968240	JX968358	JX968449	Italy	[19]
***C. cylindrospora***	**HMJAU42440**	MG250375	**OQ758203**	–	**China**	[47]; **This study**
***C. deliquescens***	**HMJAU61998**	**OP373403**	**OQ758204**	**OQ758292**	**China**	**This study**
*C. elegans*	NL-0908	JX968223	JX968341	JX968437	Sweden	[19]
*C. enderlei*	WU21272	JX968163	JX968279	–	Italy	[19]
***C. fuscimarginata***	**HMJAU45033**	**OQ780310**	**OQ758208**	**OQ758296**	**China**	**This study**
*C. fuscimarginata*	NL-3668	JX968238	JX968356	JX968448	Sweden	[19]
*C. gigasperma*	NL-3972	JX968179	JX968295	JX968403	Slovakia	[19]
*C. hausknechtii*	LE253789	JQ247194	–	–	Russia	[48]
***C. hydrophila***	**HMJAU64954**	**OQ758116**	**OQ758232**	**OQ758313**	**China**	**This study**
***C. hydrophila***	**HMJAU64955**	**OQ758117**	**OQ758233**	**OQ758314**	**China**	**This study**
***C. incarnata***	**HMJAU64968**	**OQ780316**	–	–	**China**	**This study**
*C. incarnata*	WU21897	JX968229	JX968347	JX968441	Finland	[19]
*C. incerta*	313017	NR_155031	–	–	Russia	[46]
*C. ingridiae*	WU28158	JX968244	JX968361	JX968451	Italy	[19]
*C. karakensis*	KTK05	ON392730	–	–	Pakistan	[21]
***C. lenticulospora***	**HMJAU45069**	**OQ780317**	–	–	**China**	**This study**
*C. lenticulospora*	NL-0923	JX968242	JX968359	JX968450	Sweden	[19]
*C. mesospora*	HMJAU45049	MH141419	–	–	China	[20]
*C. microrrhiza*	NL-2180	JX968222	JX968340	JX968436	Hungary	[19]
*C. moseri*	40421	MK412354	–	–	Germany	Unpublished
***C. moseri***	**HMJAU45075**	**OQ780309**	**OQ758207**	–	**China**	**This study**
***C. muscicola***	**HMJAU64939**	**OQ758113**	**OQ758223**	**OQ758309**	**China**	**This study**
***C. muscicola***	**HMJAU64940**	**OQ758115**	**OQ758224**	**OQ758310**	**China**	**This study**
***C. muscicola***	**HMJAU64941**	**OQ758114**	**OQ758225**	–	**China**	**This study**
*C. nigrescens*	WU27557	JX968234	JX968352	JX968445	Italy	[19]
*C. nitrophila*	WANG140019	KR998384	–	–	China	[49]
*C. nitrophila*	WU20916	JX968233	JX968351	JX968444	India	[19]
*C. ochrostriata* var. *favrei*	WU29786	JX968245	JX968362	JX968452	Italy	[19]
*C. olivaceopileata*	313106	NR_155028	–	–	Russia	[46]
*C. pallidospora*	WU7395	JX968239	JX968357	–	Austria	[19]
*C. parapilosella*	90551	NR_176713	–	–	Spain	[50]
***C. pilosella***	**HMJAU45062**	**OQ780305**	**OQ758205**	**OQ758294**	**China**	**This study**
***C. pilosella***	**HMJAU64957**	**OQ780306**	**OQ758206**	**OQ758295**	**China**	**This study**
***C. pilosa***	**HMJAU64947**	**OQ758122**	**OQ758222**	**OQ758307**	**China**	**This study**
***C. pilosa***	**HMJAU64948**	**OQ758123**	**OQ758221**	**OQ758308**	**China**	**This study**
***C. praticola***	**HMJAU64965**	**OQ780303**	–	–	**China**	**This study**
***C. pseudocrispa***	**HMJAU64944**	**OQ780308**	**OQ758211**	**OQ758298**	**China**	**This study**
***C. pseudocrispa***	**HMJAU64946**	**OQ780307**	**OQ758212**	**OQ758293**	**China**	**This study**
*C. pseudocrispa*	WU18009	JX968230	JX968348	JX968442	Austria	[19]
*C. pubescens*	WU20759	JX968170	JX968286	JX968396	Italy	[19]
***C. reniformis***	**HMJAU64942**	**OQ758108**	**OQ758229**	**OQ758311**	**China**	**This study**
***C. reniformis***	**HMJAU64943**	**OQ758109**	**OQ758228**	**OQ758312**	**China**	**This study**
*C. rickenii*	AH21067	MF142238	–	–	Spain	[51]
***C. romagnesii***	**HMJAU64960**	**OQ780304**	–	–	**China**	**This study**
*C. rostellata*	NL-2499	JX968162	JX968278	JX968390	Sweden	[19]
***C. rufostipes***	**HMJAU64937**	**OQ758120**	**OQ758227**	**OQ758317**	**China**	**This study**
***C. rufostipes***	**HMJAU64938**	**OQ758121**	**OQ758226**	**OQ758318**	**China**	**This study**
*C. semiglobata*	WU8794	JX968188	JX968304	JX968168	Austria	[19]
***C. siennophylla***	**HMJAU64966**	**OQ780312**	**OQ758210**	**OQ758297**	**China**	**This study**
*C. siennophylla*	NL-1210	JX968246	JX968363	JX968453	Hungary	[19]
*C. siliginea*	NL-2313	JX968225	JX968343	JX968438	Sweden	[19]
***C. singeriana***	**HMJAU64956**	**OQ780314**	**OQ758214**	–	**China**	**This study**
*C. singeriana*	WU22129	JX968166	JX968282	JX968393	Austria	[19]
***C. sinobispora***	**HMJAU64949**	**OQ758118**	**OQ758230**	**OQ758315**	**China**	**This study**
***C. sinobispora***	**HMJAU64950**	**OQ758119**	**OQ758231**	**OQ758316**	**China**	**This study**
***Conocybe* sp.1**	**HMJAU44988**	**OQ749737**	**OQ740305**	**OQ758302**	**China**	**This study**
***Conocybe* sp.1**	**HMJAU64961**	**OQ749738**	–	–	**China**	**This study**
***Conocybe* sp.2**	**HMJAU64962**	**OQ749739**	**OQ740306**	**OQ758303**	**China**	**This study**
***Conocybe* sp.2**	**HMJAU64963**	**OQ749740**	**OQ740307**	**OQ758304**	**China**	**This study**
***Conocybe* sp.3**	**HMJAU64967**	**OQ749741**	–	–	**China**	**This study**
***Conocybe* sp.4**	**HMJAU64964**	**OQ749742**	–	–	**China**	**This study**
***Conocybe* sp.5**	**HMJAU44994**	**OQ749735**	–	–	**China**	**This study**
***Conocybe* sp.5**	**HMJAU44995a**	**OQ749736**	–	–	**China**	**This study**
*C. tetrasporoides*	WU17385	JX968232	JX968350	–	New Zealand	[19]
*C. velutinomarginata*	WU28695	JX968226	JX968344	JX968439	Germany	[19]
***C. velutipes***	**HMJAU45048**	**OQ780311**	**OQ758209**	–	**China**	**This study**
*C. velutipes*	NL-2187	JX968228	JX968346	JX968440	Hungary	[19]
*C. volvicystidiata*	1212	KY346827	–	–	France	[52]
*C. watlingii*	WU22744	JX968172	JX968288	JX968398	Finland	[19]
*Descolea antarctica*	NZ5182	AF325647	–	–	USA	[53]
***D. quercina***	**HMJAU64959**	**OQ780313**	**OQ758213**	**OQ758299**	**China**	**This study**
*Pholiotina arrheni*	NL-2509	JX968261	JX968377	–	Sweden	[19]
*Ph. brunnea*	NL-1216	JX968259	JX968375	JX968461	Hungary	[19]
*Ph. coprophila*	NL-2176	JX968156	JX968273	–	Hungary	[19]
*Ph. cyanopus*	WU2134	JX968157	JX968274	JX968388	Austria	[19]
*Ph. dasypus*	NL-2279	JX968152	JX968269	JX968385	Hungary	[19]
*Ph. dentatomarginata*	NL-2921	JX968258	JX968374	JX968460	Hungary	[19]
***Ph. serrata***	**HMJAU62006**	**OP538570**	**OQ758217**	**OQ758301**	**China**	**This study**
***Ph. smithii***	**HMJAU62001**	**OP373407**	**OQ758215**	**OQ758300**	**China**	**This study**
*Ph. sulcata*	NL-1975	JX968153	JX968270	JX968386	Hungary	[19]
*Ph. teneroides*	NL-3501	JX968264	JX968379	JX968465	Slovakia	[19]
*Ph. utricystidiata*	WU20164	JX968262	JX968463	–	Germany	[19]
*Ph. vexans*	NL-3967	JX968265	JX968380	JX968466	Slovakia	[19]
*Psathyrella leucotephra*	1953	FM163226	FM160683	FM897219	Hungary	[54]
*P. piluliformis*	HMJAU37922	MG734716	MW413364	MW411001	China	[55]

Note: “–” means no relevant genetic information, and the new species are represented in bold.

## Data Availability

All the sequences have been deposited in GenBank (https://www.ncbi.nlm.nih.gov) and Mycobank (https://www.mycobank.org). The data presented in this study are deposited in the Zenodo repository, accession number https://doi.org/10.5281/zenodo.7811266.
